# MicroRNAs sequencing unveils distinct molecular subgroups of plasmablastic lymphoma

**DOI:** 10.18632/oncotarget.22219

**Published:** 2017-10-31

**Authors:** Maria Raffaella Ambrosio, Lucia Mundo, Sara Gazaneo, Matteo Picciolini, Prasad Satya Vara, Shaheen Sayed, Alessandro Ginori, Giuseppe Lo Bello, Leonardo Del Porro, Mohsen Navari, Stefano Ascani, Amhed Yonis, Lorenzo Leoncini, Pier Paolo Piccaluga, Stefano Lazzi

**Affiliations:** ^1^ Department of Medical Biotechnology, Section of Pathology, University of Siena, Siena, Italy; ^2^ Diatech Pharmacogenetics, Jesi, Italy; ^3^ Aga Khan Hospital, Kisumu, Kenya; ^4^ Aga Khan University Hospital, Nairobi, Kenya; ^5^ Pathology Unit, Ospedale Civico di Carrara, Carrara, Italy; ^6^ Research Center of Advanced Technologies in Medicine, Torbat Heydariyeh University of Medical Sciences, Torbat Heydariyeh, Iran; ^7^ Department of Experimental, Diagnostic, and Experimental Medicine, Bologna University School of Medicine, Bologna, Italy; ^8^ Euro-Mediterranean Institute of Science and Technology (IEMEST), Palermo, Italy; ^9^ Section of Pathology, Azienda Ospedaliera S. Maria di Terni, University of Perugia, Perugia, Italy; ^10^ Alexandria University, Alexandria, Egypt

**Keywords:** plasmablastic lymphoma, miRNA expression profiling, Burkitt lymphoma, extramedullary plasmacytoma, Epstein-Barr virus, Pathology section

## Abstract

Plasmablastic lymphoma (PBL) is an aggressive lymphoma, often arising in the context of immunodeficiency and associated with Epstein-Barr virus (EBV) infection. The most frequently detected genetic alteration is the deregulation of *MYC* gene through the translocation - t(8;14)(q24;q32). The diagnosis of PBL is often challenging because it has an overlap in morphology, immunophenotype, cytogenetics and virus association with other lymphomas and plasma cell neoplasms; further, its molecular basis remains elusive. In the present study we aimed to better define the possible contribution of EBV infection as well as miRNA deregulation in PBL pathogenesis.

We studied 23 cases of PBL, 19 Burkitt lymphomas (BL), and 17 extra-medullary plasmacytoma (EMPC). We used qPCR and immunohistochemistry to assess EBV latency patterns, while micro-RNA (miRNA) profiling was performed by next generation sequencing (Illumina) and validated by qPCR.

Our analysis revealed a non-canonical EBV latency program with the partial expression of some proteins characterizing latency II and the activation of an abortive lytic cycle. Moreover, we identified miRNA signatures discriminating PBL from BL and EMPC. Interestingly, based on the miRNA profile, PBL appeared constituted by two discrete subgroups more similar to either BL or EMPC, respectively. This pattern was confirmed in an independent set of cases studied by qPCR and corresponded to different clinico-pathological features in the two groups, including HIV infection, *MYC* rearrangement and disease localization.

In conclusion, we uncovered for the first time 1) an atypical EBV latency program in PBL; 2) a miRNA signature distinguishing PBL from the closest malignant counterparts; 3) the molecular basis of PBL heterogeneity.

## INTRODUCTION

Plasmablastic lymphoma (PBL) is a rare highly aggressive lymphoma that occurs most often in the context of Human Immunodeficiency Virus (HIV)-infection (40-50%), or in other settings of decreased immune surveillance [1]. Tumor cells have a well-defined morphology resembling B-immunoblasts, and express plasma cells markers [1]. However, a more or less pronounced plasmacytic differentiation as well as a variable expression of CD20 and PAX-5 can be encountered [2]. Therefore, the diagnosis of PBL is challenging because it has an overlap in cytomorphologic and immunophenotypic features with plasma cell neoplasms and with lymphomas that show blastic appearance (i.e. more immature, plasmablast-like morphology) [3]. Even using a comprehensive panel of immunostains, a confident differential diagnosis between PBL and anaplastic or plasmablastic extramedullary plasmacytoma (EMPC) is not always possible. Nonetheless, a proper distinction of these entities is mandatory as EMPC and PBL have different prognoses and currently benefit from different therapeutic strategies. On the other hand, the differential diagnosis between PBL and other lymphoma types with blastic appearance is often straightforward due to the lack of B-cell marker expression in PBL. However, BL shares numerous biologic similarities with PBL. In fact, the most frequently detected genomic alteration in PBL is t(8;14)(q24;q32) leading to *MYC/IGH* rearrangement observed in about 50% of cases [1, 3]. Other overlaps include cell morphology with the starry sky appearance, the high proliferation rate, the MYC protein over-expression and the association with EBV infection.

A common feature is the presence of the Epstein-Barr virus (EBV) DNA in tumor cells in 50 to 75% of cases, with higher frequency in HIV-positive cases [1, 3]. The molecular and genetic mechanisms underlying PBL pathogenesis remain largely unknown and are likely dependent on the complexity of biological interplays between immunodeficiency, molecular events (*MYC* gene rearrangements), co-infecting oncogenic viruses (EBV) [4] and chronic immune activation (expression of immune-checkpoint proteins) [5]. It is unknown, however, whether *MYC* translocation represents the initiating or a late genetic event in PBL pathogenesis, and furthermore the latency program of EBV in neoplastic cells is still debated. In fact, although the type I latency is commonly detected, type III latency can be observed in patients with HIV infection and in those patients with post-transplant PBL [3].

In the present study we aimed to better define the pathogenesis and molecular features of PBL by 1) assessing the EBV latency patterns in PBL by qPCR and immunohistochemistry; 2) performing, for the first time to the best of our knowledge, an unbiased miRNA profiling of PBL, EMPC, and BL by next generation sequencing (NGS).

## RESULTS

### qPCR and immunohistochemistry reveal a non-canonical latency type and an abortive lytic cycle of EBV in plasmablastic lymphoma

**Figure 1:**
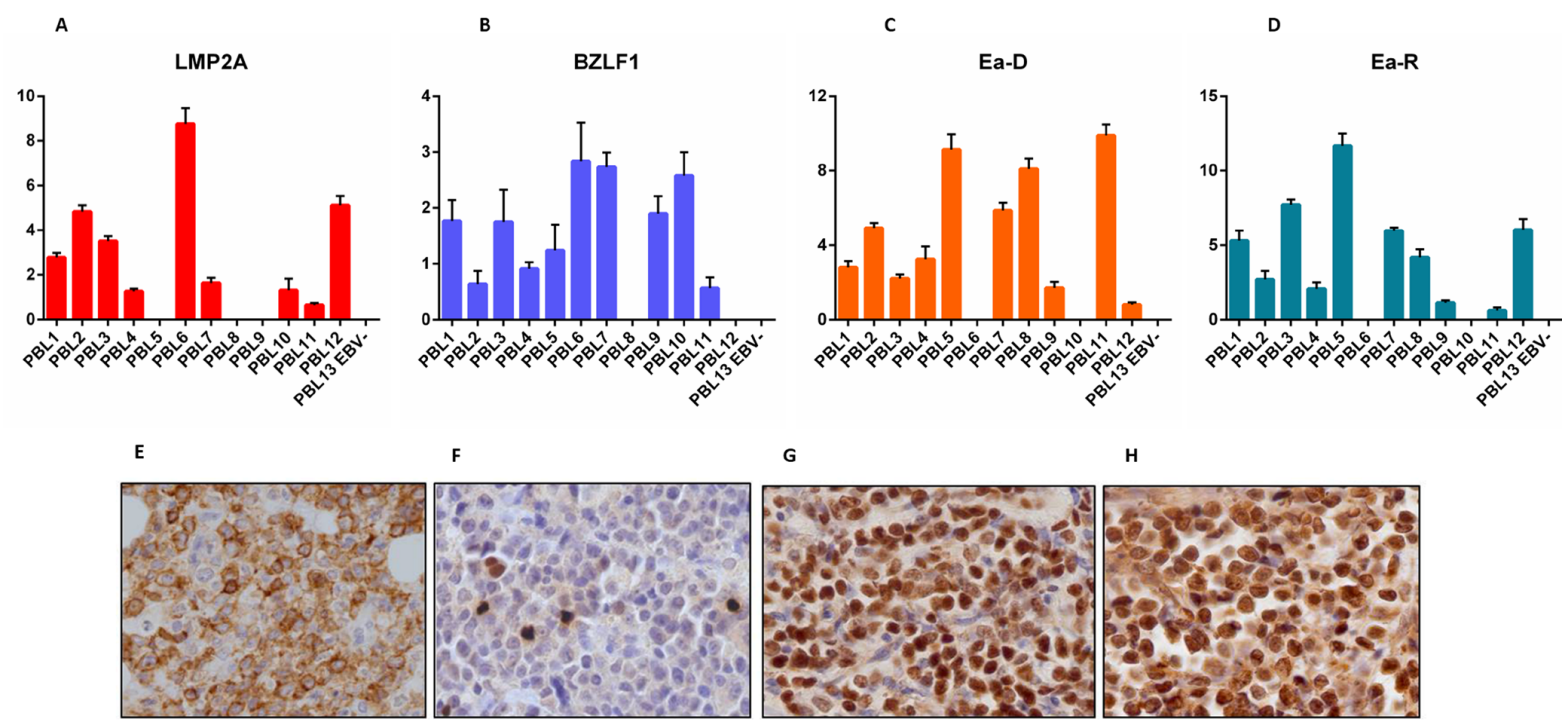
RT-qPCR and immunohistochemistry reveal a non-canonical latency type and an abortive lytic cycle of EBV in neoplastic cells *LMP-2A* **A.**, **E.**, *BZLF-1/*ZEBRA **B.**, **F.**, *BMRF-1*/Ea-D **C.**, **G.**, *BHRF-1*/Ea-R **D.**, **H.**, expression by RT-qPCR (across the 13 PBL cases) and immunohistochemistry (in one exemplificative PBL case; original magnification, 40x). In qPCR graphs, y-axis represents gene expression values (2 Delta Ct formula).

We checked the expression of *EBNA-1, EBNA-2, EBNA-3, LMP-1, LMP-2A*, EBER transcripts, *BZLF-1/*ZEBRA, *BMRF-1*/Ea-D, *BHRF-1*/Ea-R, *BLLF1*/gp350 across the 13 PBL cases (12 EBV-positive and 1 EBV-negative). EBNA-1 was detected in 12/13 cases (92.3%), LMP-1 and LMP-2 in 1/13 and 10/13 (7.7% and 76.9%, respectively) (Figure 1), EBNA-2 and EBNA-3 in 0/13, and EBER in 12/13 (92.3%). *BZLF-1/*ZEBRA was found in 12/13 cases (92.3%) and both *BMRF-1*/Ea-D e *BHRF-1*/Ea-R in 10/13 (76.9%) (Figure 1), while none of the cases expressed BLLF1/gp350 (Figure 1).

We then determined the expression of all the corresponding encoded-proteins. Immunohistochemistry (IHC) confirmed the non-canonical latency associated program with the partial expression of some proteins characterizing latency II (i.e. LMP-1 in 1/13 and LMP-2 in 6/13 of the cases) (Figure 1). There was also the expression of proteins characterizing the lytic phase of the virus (i.e. BZLF-1/ZEBRA in 12/13, and BHRF-1/Ea-R and BMRF-1/Ea-D in 9/13 of the cases) (Figure 1). BLLF1/gp350 was not detected in any case. The qPCR data substantially corresponded to those obtained by IHC; however, slight differences emerged likely due to the higher sensitivity of qPCR.

The same analyses were then carried out in the validation cohort with substantially identical results (Figure 1; Table 3).

**Table 1:** Clinical, histologic, immunophenotypic and FISH features of discovery cohort patients

	Discovery cohort			Validation cohort		
	PBL	EMPC	BL	PBL	EMPC	BL
**Mean age (range), years**	54.2 (28-76)	65.2 (44-76)	17.7 (3-40)	66.3 (45-87)	59 (45-74)	18.3 (5-43)
**Male:female ratio**	10/3	5/2	4/5	7/3	5/2 (3NA)	3/6
**Oral**	5	1	1	1	1	1
**Extra-Oral**	8 (vulva, ileum, pleura, penis, skin)	6 (stomach, pleura, chest-wall, scrotum, vertebra)	8 (lymph-nodes, stomach, ileum, uterus, ovary)	9 (Ileum, stomach, thyroid, chest-wall, lymph-node, colon)	9 (iliac bone, chest-wall, lymph-node, vertebra)	8 (ileum, stomach, lymph-nodes)
**Morphology**	blastic *versus* more pronounced plasmacytic differentiation; necrosis, mitotic figures and apoptotic bodies with a “starry-sky” appearance	proliferation of mature plasma cells; 3 cases more blastic appearance, 1 case anaplastic	medium-sized cells, with monotonous and uniform appearance, squared-off cytoplasm, cohesive growth and starry-sky appearance	blastic *versus* more pronounced plasmacytic differentiation; necrosis, mitotic figures and apoptotic bodies with a “starry-sky” appearance	proliferation of mature plasma cells; 3 cases more blastic appearance, 1 case anaplastic	medium-sized cells, with monotonous and uniform appearance, squared-off cytoplasm, cohesive growth and starry-sky appearance
**Immunophenotype**						
CD38	13	9	9	10	10	9
CD138	13	9	0	10	10	-
IRF4/MUM1	13	-	0	10	-	-
BCL2	13 (weak)	-	0	10 (weak)	-	0
BCL6	13 (weak)	0	9	10 (weak)	-	9
CD10	13 (weak)	0	9	10 (weak)	-	9
CD20	0	0	9	0	0	9
PAX5	0	0	9	0	0	-
HHV8	0	-	-	2	-	-
ALK1	0	-	-	0	-	-
CD56	1	-	-	-	-	-
MYC (20-50%)	0	2	0	0	-	0
MYC (>50%)	5	0	0	7	-	0
MYC (>80%)	8	0	9	2	-	9
***MYC*** **translocation (FISH)**	8/13	2/7	9/9	9/10	2/10	9/9
**EBV infection**	12/13	2/7	5/9	4/10	0/10	6/9
**Immune suppression**	7/13	NA	NA	2/10	NA	NA

**PBL:** plasmablastic lymphoma; **EMPC:** extramedullary plasmacitoma; **BL:** Burkitt lymphoma; **IHC:** immunohistochemistry; **pos.:** positivity; **neg.:** negativity; **NA.:** not available.

**Table 2:** Primers’ sets used for qPCR analysis of EBV-encoded genes

Assay	Primers set
*EBNA1*	5’- TAC AGG ACC TGG AAA TGG CC- 3’
	5’- TCT TTG AGG TCC ACT GCC G-3’
*EBNA2*	5’-TAA CCA CCC AGC GCC AAT C-3’
	5’- GTA GGC ATG ATG GCG GCA G 5’
*EBNA3c*	5’- CTG GCA AAA CTT GCT CCA- 3’
	5’- GTG CTT CTG CCT TAT CAG A- 3’
*BHRF1*	5’-AGA AAC ACC TCT CCG CCT TT-3’
	5’- ATC CAC ATG TTC GGT GTG TG-3’
*LMP1*	5’- CAG TCA GGC AAG CCT ATG A-3’
	5’- CTG GTT CCG GTG GAG ATG A-3’
*BMRF1*	5’- CAACACCGCACTGGAGAG- 3’
	5’- GCCTGCTTCACTTTCTTGG- 3’
*LMP2A*	5’- AGC TGT AAC TGT GGT TTC CAT GAC-3’
	5’- GCC CCC TGG CGA AGA G-3’
*BLLF1*	5'-ATCCAGTTGTATTCAAGGTAGG-3'
	5'-ACTCATTATCACACGAACGG-3'
*BZLF1*	5’- AAA TTT AAG AGA TCC TCG TGT AAAACA TC-3’
	5’- CGC CTC CTG TTG AAG CAG AT-3’
*HPRT*	5’-AGC CAG ACT TTG TTG GAT TTG-3’
	5’-TTT ACT GGC GAT GTC AAT AAG-3’

**Table 3:** qPCR and immunohistochemical latency results on validation cohort

	RT-qPCR		IHC/ISH	
	Positive cases (n.)	Negative cases (n.)	Positive cases (n.)	Negative cases (n.)
**EBNA-1**	4	6	n.p.	n.p.
**EBNA-2/3**	0	10	n.p.	n.p.
**LMP-1**	0	10	0	10
**LMP-2**	4	6	3	7
**EBER**	-	-	4	6
**ZEBRA**	3	7	4	6
**EaR**	4	6	3	7
**EaD**	4	6	3	7
**gp350**	0	10	0	10

**ISH:** in situ hybridization; **n.:** number of cases; **n.p.:** not performed

Together, these data indicate a non-canonical EBV-latency pattern in PBL.

### Plasmablastic lymphomas can be divided into two groups based on the miRNA profiling

**Figure 2:**
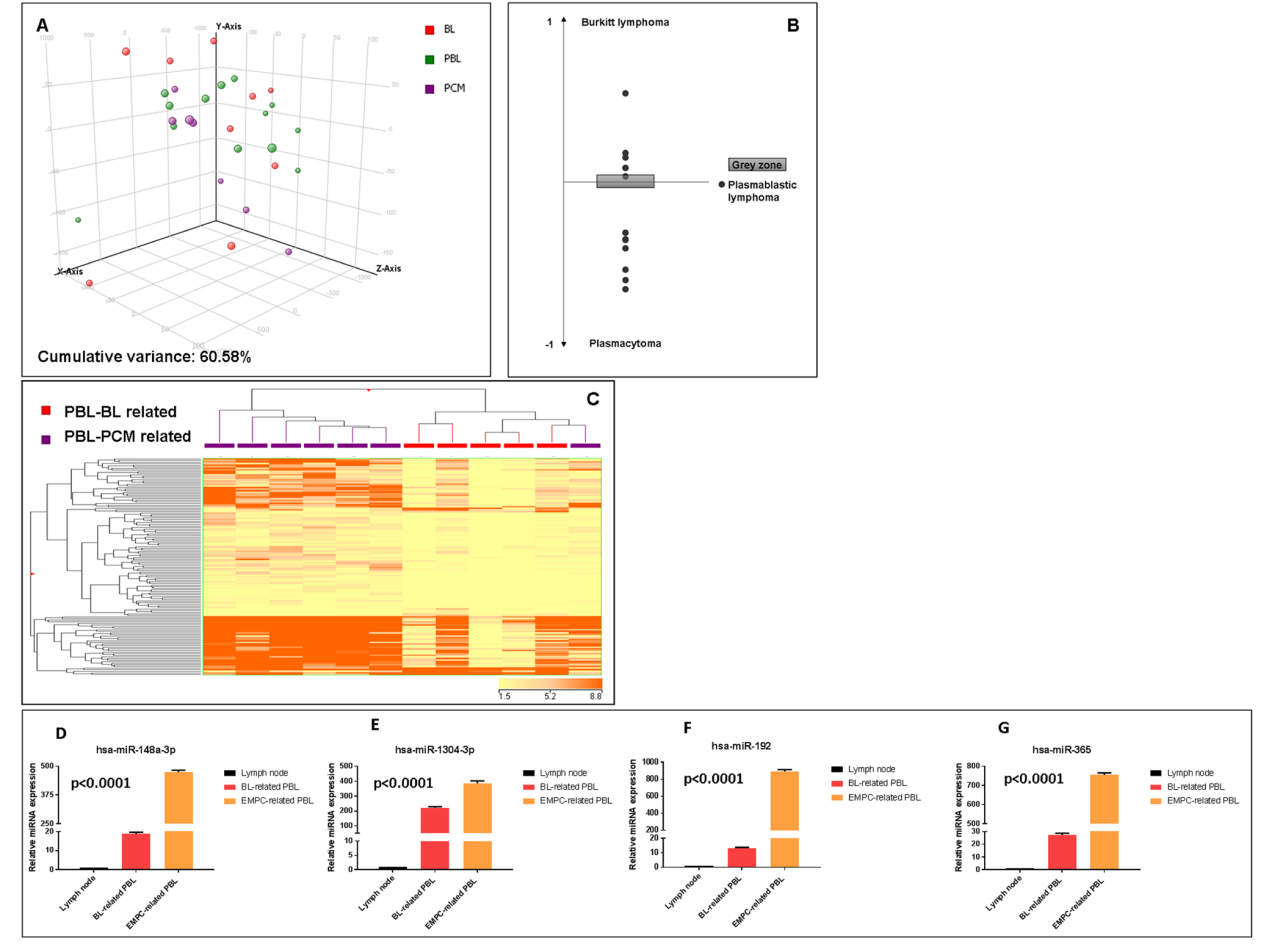
Plasmablastic lymphomas include two molecular subgroups related to either EMPC or BL Principal component analysis of plasmablastic lymphoma (PBL), extramedullary plasmacytoma (EMPC) and Burkitt lymphoma (BL) cases indicated a basic relation between the three entities at miRNA level **A.**. A cell type classifier based on a support vector machine algorithm was generated to discriminate BL and EMPC based on the expression of differentially expressed miRNA. When the algorithm was applied to PBL, they were classified as either BL or EMPC indicating their heterogeneity reflecting the similarity to either one of the other two tumor types (BL and EMPC) **B.**. Grey zone represent value below significance (0.05). Supervised analysis (*t*-test) comparing BL-related *versus* EMPC-related PBLs identified a series of differentially expressed genes **C.**. In the matrix, the dendrogram was generated using a hierarchical clustering algorithm based on the average-linkage method. In the matrix, each column represents a sample and each row represents a gene. The colour scale bar shows the relative gene expression changes normalized by the standard deviation (0 is the mean expression level of a given gene). Differential expression of hsa-miR-192-5p, hsa-miR-1304-3p, hsa-miR-148a-3p and hsa-miR-365-3p in BL-related *versus* EMPC-related PBLs group **D.**-**G.** Values are expressed as mean ± standard deviation (ST). Statistical significance was determined with Student's unpaired *t*-test (*p* < 0.005).

First, we sought to investigate the relation of PBL with BL and EMPC as far as miRNA expression was concerned. When an unsupervised approach was used, namely PCA, PBL appeared relatively heterogeneous and was not clearly distinct from BL and EMPC, indicating some degree of similarity with both of them. To better determine the possible relation with these two tumors, we developed a miRNA expression-based cell type classifier to establish whether single PBL cases could be related to either one (or none) of the two (BL or EMPC). We found that 7 out of 13 cases were significantly closer to EMPC, 5 out of 13 to BL, and 1 was not clearly classified (Figure 2A-B, Table 4). By a supervised analysis, we identified 136 cellular miRNA differentially expressed in the two new subgroups (Figure 2C; Table 5a and 5b): 123 were up-regulated in “EMPC-related” PBLs, while 13 were up-regulated in “BL-related” ones. Among others, the expression level of hsa-mir-192-5p, hsa-mir-1304-3p, hsa-mir-148a-3p, hsa-miR-365-3p was studied by RT-qPCR as technical validation. We observed that these miRNA were up-regulated in the cases belonging to the EMPC-related cluster and down-regulated in the BL-related samples, confirming NGS results (Figure 2D). Interestingly, the two groups of PBLs tended to differ also for some clinical features. The patients BL-related were younger (mean age: 50.2 years), with a slight male predominance (M:F= 3:2), an extra-oral localization and a blastic appearance; the majority of them (4 out 5) were HIV-negative. The patients EMPC-related were older (mean age: 56.85 years), mainly male (M:F= 6:1), with an oral localization (4 out 7) and a more pronounced plasmacytic differentiation; the majority of them (4 out 7) were HIV-positive. Of note, 5/5 BL-related cases presented with *MYC* rearrangements at FISH, while only 2/7 EMPC presented with such feature (fisher exact test, p=0.03).

**Table 4:** Correlation between PBL and BL or EMPC

Identifier	Cell type	Predicted	Confidence Measure
H122 (raw)	PBL	BL	0.09583959
V109 (raw)	PBL	BL	0.10874487
s838 (raw)	PBL	BL	0.1592613
s613 (raw)	PBL	BL	0.20450206
s806 (raw)	PBL	BL	0.23559976
s1805 (raw)	PBL	EMPC	0.27476195
H276 (raw)	PBL	EMPC	0.45076987
s1529 (raw)	PBL	EMPC	0.4700034
s13725 (raw)	PBL	EMPC	0.47044697
s11224 (raw)	PBL	EMPC	0.5045746
s189 (raw)	PBL	BL	0.59934336
H333 (raw)	PBL	EMPC	0.6791907
V376 (raw)	PBL	EMPC	0.95444125

**Table 5A:** miRNA differentially expressed in the two PBL groups (BL-related *versus* EMPC-related)

miRNA	p	Fold change	Regulation in BL-related group
hsa-let-7a-5p	0.028575	256	down
hsa-let-7b-5p	0.006063	10809.406	down
hsa-let-7d-3p	0.044231	2.6918004	down
hsa-let-7d-5p	0.012786	3.482202	down
hsa-let-7e-5p	0.028014	4.3297553	down
hsa-let-7f-5p	0.018527	159.15468	down
hsa-let-7g-5p	0.046021	13.387893	down
hsa-miR-101-3p	0.002134	48.502926	down
hsa-miR-103a-3p	0.004657	21.5344	down
hsa-miR-103b	0.011111	18.74678	down
hsa-miR-106b-3p	0.02385	4.594794	down
hsa-miR-106b-5p	0.025859	13.125367	down
hsa-miR-10b-5p	0.045394	1.17E+07	down
hsa-miR-125a-5p	0.022511	40.58451	down
hsa-miR-125b-1-3p	0.002118	2.6918004	down
hsa-miR-126-3p	0.012516	128	down
hsa-miR-126-5p	0.0226	159.15468	down
hsa-miR-127-3p	0.030994	86.13763	down
hsa-miR-128-3p	0.00253	8.659511	down
hsa-miR-1304-3p	0.039182	5.0730634	down
hsa-miR-130a-3p	0.015367	5.0730634	down
hsa-miR-130b-3p	0.024846	5.278032	down
hsa-miR-132-3p	0.003019	3.8446627	down
hsa-miR-139-5p	0.037432	2.122426	down
hsa-miR-140-3p	0.004751	436.9814	down
hsa-miR-140-5p	0.038232	2.164878	down
hsa-miR-143-3p	0.023024	40743.586	down
hsa-miR-145-3p	0.034978	4.780439	down
hsa-miR-145-5p	0.019368	26.250734	down
hsa-miR-146a-5p	0.002346	111.43049	down
hsa-miR-146b-5p	0.003099	1176.267	down
hsa-miR-148a-3p	0.003072	1.38E+10	down
hsa-miR-148b-3p	0.001742	13.387893	down
hsa-miR-151a-3p	0.017852	49.47307	down
hsa-miR-155-5p	0.042635	62.74499	down
hsa-miR-15a-5p	0.01427	4.9735837	down
hsa-miR-15b-5p	0.029421	3.0920668	down
hsa-miR-16-5p	0.002014	5967.2734	down
hsa-miR-181a-3p	0.00621	2.390219	down
hsa-miR-181a-5p	0.003179	26353.643	down
hsa-miR-181b-5p	0.028867	218.4907	down
hsa-miR-181c-5p	0.024008	5.0730634	down
hsa-miR-182-5p	0.041277	141.3235	down
hsa-miR-183-5p	0.017472	34.638054	down
hsa-miR-186-5p	0.006628	182.82076	down
hsa-miR-191-5p	0.003595	6854.601	down
hsa-miR-192-5p	0.003457	10.146127	down
hsa-miR-193b-3p	0.003606	179.23567	down
hsa-miR-195-5p	0.037465	3.3469734	down

**Table 5B:** miRNA differentially expressed in the two PBL groups (BL-related *versus* EMPC-related)

miRNA	p	Fold change	Regulation in BL-related group
hsa-miR-196a-5p	0.033883	9.373392	down
hsa-miR-197-3p	0.02192	13.387893	down
hsa-miR-199a-3p	0.043553	8.489703	down
hsa-miR-210-3p	0.020199	5.1745324	down
hsa-miR-2110	0.020158	2	down
hsa-miR-212-3p	0.037432	2.122426	down
hsa-miR-21-3p	0.027591	25.73597	down
hsa-miR-214-3p	0.016095	5.1745324	down
hsa-miR-21-5p	0.016598	1.60E+09	down
hsa-miR-221-3p	0.009039	194.0117	down
hsa-miR-221-5p	0.019567	3.482202	down
hsa-miR-222-3p	0.001477	120.616684	down
hsa-miR-22-3p	0.021866	144.15012	down
hsa-miR-23a-3p	0.008328	48.502926	down
hsa-miR-23b-3p	0.03084	48.502926	down
hsa-miR-24-3p	0.047314	13.655676	down
hsa-miR-25-3p	4.42E-04	324.6761	down
hsa-miR-26a-5p	0.006917	8693.463	down
hsa-miR-26b-5p	8.58E-04	93.23874	down
hsa-miR-27a-3p	0.011841	1176.267	down
hsa-miR-27b-3p	0.011343	512	down
hsa-miR-27b-5p	0.048211	2	down
hsa-miR-28-3p	0.001606	50.4626	down
hsa-miR-28-5p	7.68E-04	6.0628657	down
hsa-miR-29b-3p	0.012198	5.6011167	down
hsa-miR-29c-3p	0.004106	7.3907156	down
hsa-miR-29c-5p	0.001043	3.482202	down
hsa-miR-301a-3p	0.00456	4.504692	down
hsa-miR-3074-5p	0.00282	5.3836007	down
hsa-miR-30a-5p	0.028991	70.66175	down
hsa-miR-30b-5p	0.006546	7.1037035	down
hsa-miR-30c-5p	0.003975	15.686253	down
hsa-miR-30d-5p	0.020276	86.13763	down
hsa-miR-30e-3p	0.009217	5.6011167	down
hsa-miR-30e-5p	0.012316	231.86519	down
hsa-miR-3184-3p	0.036321	3.8446627	down
hsa-miR-3184-5p	0.047828	29.562862	down
hsa-miR-324-5p	0.007311	2.8565736	down
hsa-miR-326	0.017578	2.6918004	down
hsa-miR-328-3p	0.040136	3.6228948	down
hsa-miR-340-5p	0.029992	2.6918004	down
hsa-miR-345-5p	3.51E-05	5.3836007	down
hsa-miR-34a-5p	0.025859	13.125367	down
hsa-miR-3613-5p	0.017127	3.2169964	down
hsa-miR-361-3p	0.018605	9.009385	down
hsa-miR-3615	0.006715	6.562682	down
hsa-miR-361-5p	0.011483	5.7131467	down
hsa-miR-365a-3p	0.024084	2.3433483	down
hsa-miR-374a-5p	0.001147	2.4867918	down
hsa-miR-374b-5p	7.10E-04	3.1539128	down
hsa-miR-378a-3p	0.010879	59.125725	down
hsa-miR-378d	0.007875	3.1539128	down
hsa-miR-381-3p	0.015433	2.122426	down
hsa-miR-423-3p	0.01819	64	down
hsa-miR-425-5p	0.025742	10.146127	down
hsa-miR-4448	0.043389	18.01877	down
hsa-miR-454-3p	0.008394	5.0730634	down
hsa-miR-4633-5p	0.030738	49.47307	down
hsa-miR-497-5p	0.008057	4.244852	down
hsa-miR-500a-3p	0.01973	3.0920668	down
hsa-miR-502-3p	0.015503	3.031433	down
hsa-miR-574-3p	0.018238	3.9215622	down
hsa-miR-615-3p	0.032965	3.1539128	down
hsa-miR-625-3p	0.016429	7.390717	down
hsa-miR-629-5p	0.012786	3.482202	down
hsa-miR-652-3p	0.037863	7.843124	down
hsa-miR-654-3p	0.042732	2.3433483	down
hsa-miR-660-5p	0.023198	2.5365317	down
hsa-miR-664a-3p	0.007032	2.4867918	down
hsa-miR-6737-5p	0.028424	2.0400033	down
hsa-miR-92b-3p	0.004056	1747.9257	down
hsa-miR-93-5p	0.014503	56.829643	down
hsa-miR-96-5p	0.027386	3.482202	down
hsa-miR-98-5p	0.037122	3.4139185	down
hsa-miR-3178	0.025964	66.58581	up
hsa-miR-3648	0.010903	4.080006	up
hsa-miR-4492	0.037656	91768.664	up
hsa-miR-4497	0.029851	3859.7363	up
hsa-miR-4508	0.039168	1108.4174	up
hsa-miR-5787	0.01597	22492.303	up
hsa-miR-6087	0.032981	14.207409	up
hsa-miR-6126	0.001043	12.125731	up
hsa-miR-663b	0.019219	5.6011167	up
hsa-miR-6739-5p	1.33E-04	3.482202	up
hsa-miR-6743-5p	0.029317	4.594794	up
hsa-miR-7704	0.026789	62990.94	up
hsa-miR-8072	0.034227	2.0808065	up

When we applied the cell classifier based on the expression of hsa-mir-192-5p, hsa-mir-1304-3p, hsa-mir-148a-3p, hsa-miR-365-3p to an independent set of 10 cases, we found that 3/9 cases were classified as EMPC-related and 6/9 as BL-related, one case not being evaluable for technical reasons (qPCR failure) (Supplementary Figure 1). Noteworthy, training and test set were comparable concerning HIV infection (6/13 vs 2/10, Fisher exact test, p=0.09)

### Plasmablastic lymphomas and plasmacytomas have different miRNA profiles

**Figure 3:**
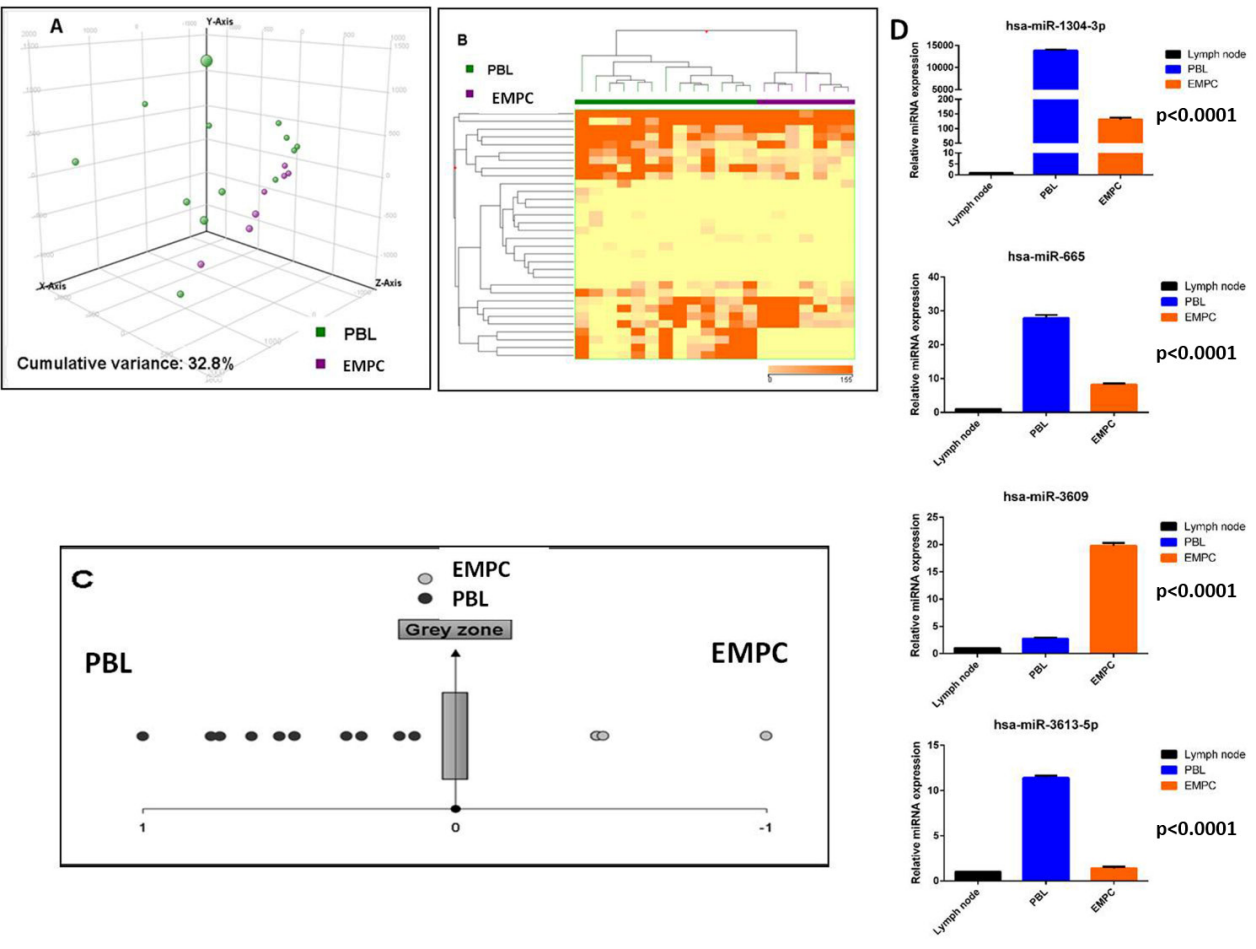
Plasmablastic lymphoma and extramedullary plasmacytoma are distinct based on the miRNA profile Principal component analysis of plasmablastic lymphoma (PBL) and extramedullary plasmacytoma (EMPC) cases **A.**. Supervised analysis (*T*-test) comparing PBL and EMPC cases identified a series of differentially expressed genes **B.**. In the matrix B, the dendrogram was generated using a hierarchical clustering algorithm based on the average-linkage method. In the matrix, each column represents a sample and each row represents a gene. The colour scale bar shows the relative gene expression changes normalized by the standard deviation (0 is the mean expression level of a given gene). A cell type classifier based on a support vector machine algorithm was generated to discriminate PBL and EMPC based on the expression of differentially expressed miRNA **C.**. Grey zone represent value below significance (0.05). Differential expression of hsa-miR-665, hsa-miR-3613-5p, hsa-miR-1304-3p and hsa-miR-3609 in PBL *versus* EMPC group **D.**. Values are expressed as mean ± standard deviation (ST). Statistical significance was determined with Student's unpaired *t*-test (*p* < 0.005).

In order to unveil specific differences between the PBL and EMPC, we compared the two diseases in term of miRNA expression. When unsupervised approach was adopted, both principal component analysis (PCA) and hierarchical clustering (HC) failed to clearly separate the two entities (data not shown). Then, by supervised analysis, we identified 48 differentially expressed miRNA, including 38 EBV-encoded and 10 cellular miRNA which could clearly differentiate the two groups when PCA and HC methods were applied (Figure 3A-3B, Supplementary Table 2). Of note, a linear discriminant analysis indicated that as few as four miRNA (miR-665, miR-3613-5p, miR-1304-3p and miR-3609) could correctly discriminate the samples. Accordingly, based on the expression of such 4 miRNA, we generated a cell classifier and applied it to our series, being able to correctly classify all cases (overall accuracy 100%; Table 6; Figure 3C). The expression of these four miRNA was also validated by RT-qPCR in the same panel of cases, confirming NGS results (Supplementary Figure 2). Furthermore, for the potential diagnostic implication, we studied their expression by qPCR in an independent validation cohort, consisting of 20 cases (10 PBL and 10 EMPC). When the cell classifier was applied, 9/10 EMPC and 10/10 PBL were correctly identified (95% overall accuracy; Supplementary Figure 2; Table 7). All the three anaplastic EMPC included in the analysis were correctly classified.

**Table 6:** Molecular diagnosis of PBL *versus* EMPC: diagnostic accuracy of the 4-miRNA based classifier in the discovery set

		Conventional histopathology	
		PBL	EMPC
**miRNA profiling**	*PBL*	13	0
	*EMPC*	0	7
			
		**Value**	**95% CI**
**Accuracy**	**SP**	100%	100-100
	**ST**	100%	100-100
	**PPV**	100%	100-100
	**NPV**	100%	100-100
	**LR+**	10	1.56 to 64.20
	**LR-**	NC	NC
	**Overall accuracy**	100% (20/20)	
	**Pre-test probability**	50%	28-72

**Table 7:** Molecular diagnosis of PBL *versus* EMPC: diagnostic accuracy of the 4-miRNA based classifier in the validation set

		Conventional histopathology	
		PBL	EMPC
**miRNA profiling**	*PBL*	10	1
	*EMPC*	0	9
			
		**Value**	**95% CI**
**Accuracy**	**SP**	90%	71-100
	**ST**	100%	100-100
	**PPV**	91%	74-100
	**NPV**	100%	100-100
	**LR+**	10	1.56 to 64.20
	**LR-**	NC	NC
	**Overall accuracy**	95% (19/20)	
	**Pre-test probability**	50%	28-72

### Plasmablastic lymphomas and Burkitt lymphomas have different miRNA profiles

**Figure 4:**
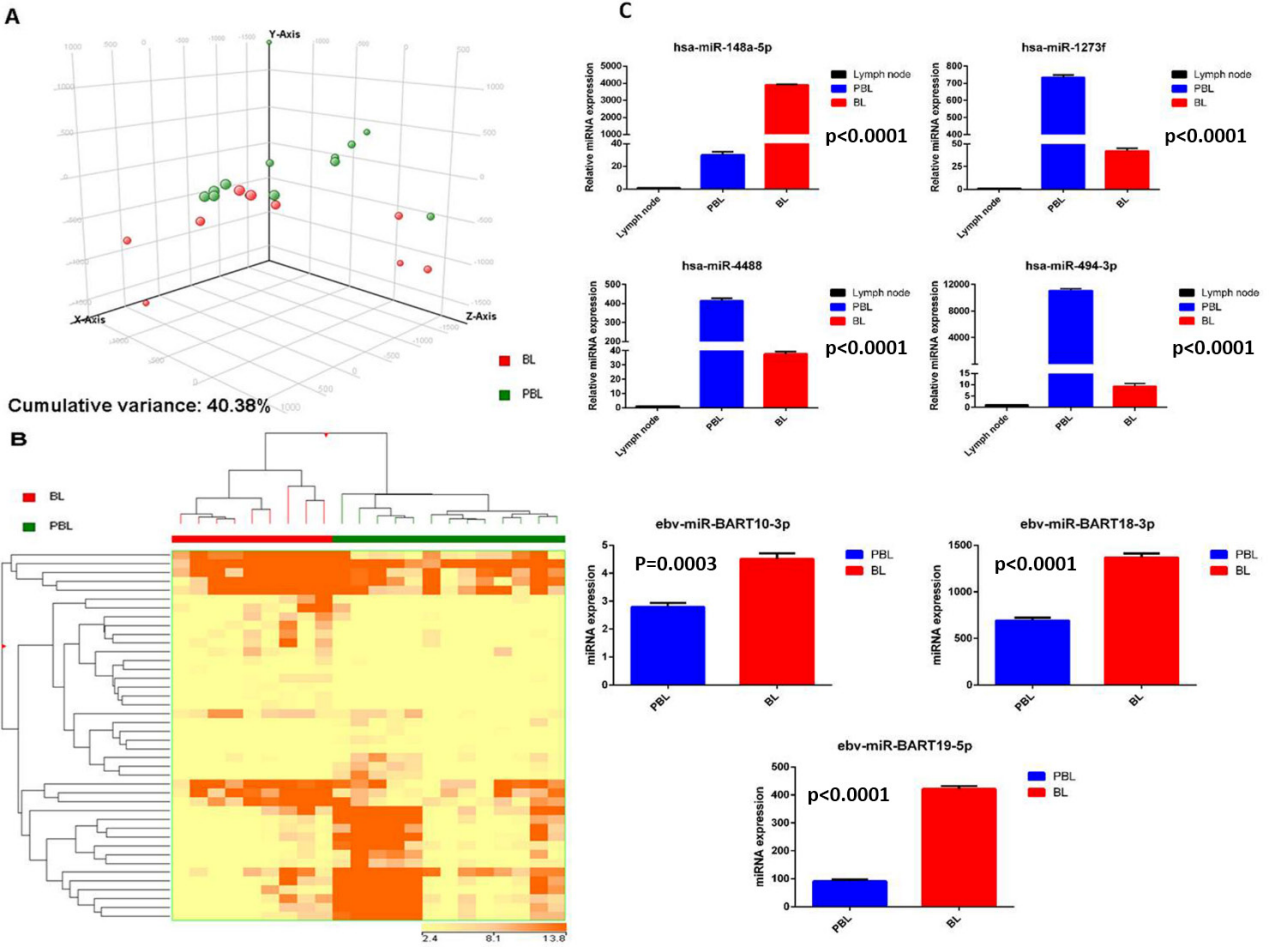
Plasmablastic lymphoma and Burkitt lymphoma are distinct based on the miRNA profile Principal component analysis of plasmablastic lymphoma (PBL) and Burkitt lymphoma (BL) cases **A.**. Supervised analysis (*t*-test) comparing PBL and BL cases identified a series of differentially expressed genes **B.**. In the matrix B, the dendrogram was generated using a hierarchical clustering algorithm based on the average-linkage method. In the matrix, each column represents a sample and each row represents a gene. The colour scale bar shows the relative gene expression changes normalized by the standard deviation (0 is the mean expression level of a given gene). Cellular miRNA (hsa-miR-1273f, hsa-miR-148a-5p, hsa-miR-4488, hsa-miR-494-3p) and EBV-viral miRNA (EBV-miR-Bart10-3p, EBV-miR-18-3p, EBV-miR-19-5p) expression in PBL *versus* BL cohort groups **C.**. Values are expressed as mean ± standard deviation (ST). Statistical significance was determined with Student's unpaired *t*-test (*p* < 0.005).

We then compared PBL to BL. By PCA the two diseases appeared relatively similar, without clear distinction (Figure 4A). By supervised analysis, we could identify a series of 42 differentially expressed miRNA (23 cellular and 19 EBV miRNA) (Figure 4B, Supplementary Table 3). As technical validation, some of them (hsa-miR-148-5p, hsa-miR-1273f, hsa-miR-4488, hsa-miR-494-3p; EBV-miR-BART10-3p, EBV-miR-BART18-3p and EBV-miR-BART19-5p) were also evaluated by qPCR, that again confirmed NGS data (Figure 4C). With the exception of miR-148a-5p, all cellular miRNA were up-regulated in PBL cases in respect to BL samples; a higher expression of all viral miRNA was detected in BL in comparison to PBL group.

These findings were also confirmed in our validation cohort (Supplementary Figure 3).

### Plasmablastic lymphomas differ in the miRNA profile based on HIV infection

**Figure 5:**
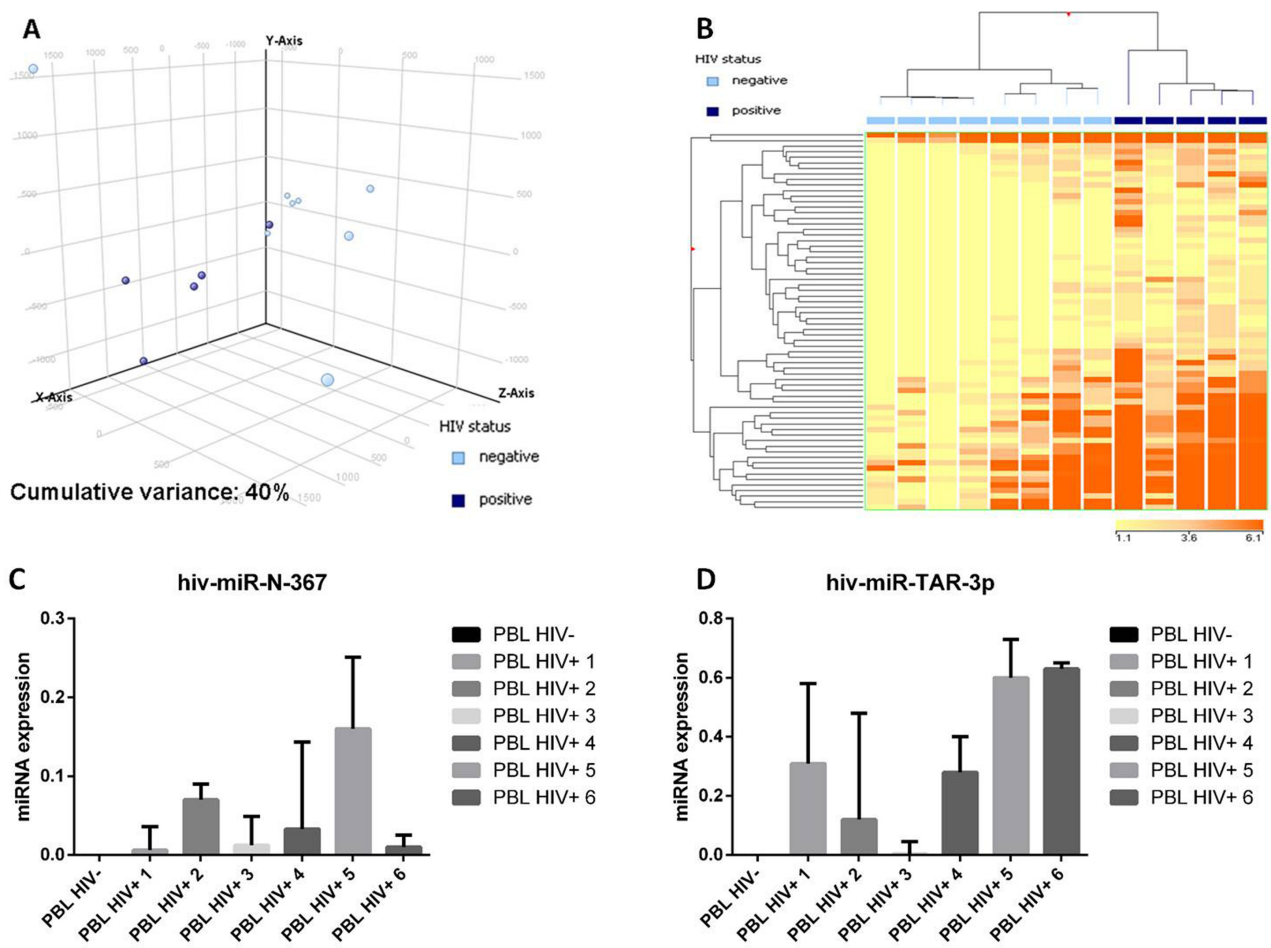
Plasmablastic lymphomas differ in their miRNA profile according to the presence of HIV Principal component analysis of HIV-positive and HIV-negative plasmablastic lymphomas (PBLs) indicated a rough distinction of the two groups based on miRNA expression **A.**. Supervised analysis (*T*-test) comparing HIV-positive *versus* HIV-negative PBLs identified a series of differentially expressed genes **B.**. In the matrix, the dendrogram was generated using a hierarchical clustering algorithm based on the average-linkage method. In the matrix, each column represents a sample and each row represents a gene. The colour scale bar shows the relative gene expression changes normalized by the standard deviation (0 is the mean expression level of a given gene). 5C. HIV-miR-367 expression in HIV positive and HIV negative PBL cases **C.**. HIV-miR-TAR-3p expression in HIV positive and HIV negative PBL cases **D.**.

Since the association with HIV might affect PBL miRNA profiling, we directly compared HIV-positive *versus* HIV-negative cases. Unsupervised evaluation by PCA was already able to distinguish the two groups (Figure 5A). Further, the supervised analysis identified as many as 68 differentially expressed miRNA all of which were of cellular origin and were up-regulated in the HIV-positive group (Supplementary Table 4). By contrast, no EBV-encoded miRNA was differentially expressed. Based on the expression of such 68 miRNA, the cases were clearly distinct at HC (Figure 5B). None of the so far known HIV encoded miRNA emerged as significantly discriminating the two groups at NGS analysis. Therefore, in order to exclude possible underestimation of HIV-encoded miRNA contribution, we used a more sensitive qPCR approach to investigate this issue further. Although at low level, we found that both the tested HIV-encoded miRNA (HIV-miR-N-367 and HIV-miR-TAR-3p) were expressed in HIV cases but not in the negative ones (Figure 5C-5D).

### Key cellular signaling pathways and metabolic activities may be affected by deregulated cellular and viral miRNA

To explore the potential involvement of the detected miRNA in PBL pathogenesis, we searched for genes targeted by the miRNA discriminating the molecular subtypes of PBL that were previously confirmed as direct targets biochemically. Among others, we found genes encoding for tumor suppressors (*PTEN, PBX2*) and lipid metabolism controller (*PPARGC1A, PLIN2*/adipophilin) whose expression levels were found to be regulated by BART-19-5p, and *FOXK2*, a gene involved in the control of viral and cellular promoter elements, regulated by hsa-miR148a-5p, has-miR-3609, has-miR-365.

**Figure 6:**
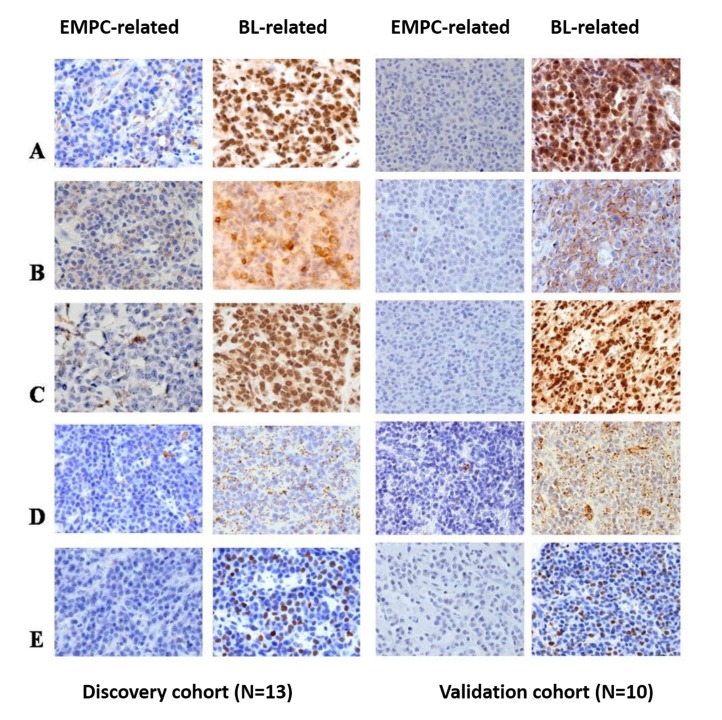
PTEN, PBX2, PPARGC1A, PLIN2/adipophilin, and FOXK2 protein expression in the two sub-groups of PBL PTEN, PBX2, PPARGC1A, PLIN2/adipophilin and FOXK2 showed lower level of expression in all the PBL cases that clustered closer to the EMPC cases than to the BL cases; one representative case for each sub-group is depicted. **A.**-**E.**, O.M.: 40x. PBL: plasmablastic lymphoma; EMPC: extramedullary plasmacytoma; BL: Burkitt lymphoma.

The expression level of the encoded proteins was then checked by immunohistochemistry in 13 PBL cases. BL-related cases presented with significantly higher degree of expression of PTEN, PBX2, PPARGC1A, PLIN2/adipophilin and FOXK2 (Figure 6A-6E and) (p<0.05).

We also investigated the potential pathobiological relevance of these genes by gene set enrichment analysis (GSEA). We found several gene sets significantly enriched; among others those associated to Kruppel-like factor 1 (*KLF1*), enhancer of zeste 2 polycomb repressive complex 2 subunit (*EZH2*) and *MYC* appeared of particular interest due to the high scores and consistent recurrence (Supplementary Table 5).

### Novel miRNA discovery

We analyzed 13 PBL miRNA-seq libraries to identify candidate novel miRNA species that were deregulated in this tumor type. After sequence filtering, we enumerated 133 candidate novel miRNA, not identified in miRBase v21 (Supplementary Table 6). Differential expression analysis was carried out between PBL *versus* EMPC and PBL *versus* BL. We found 27 miRNA differentially expressed between PBL and BL, and 7 between PBL and EMPC (Supplementary Tables 7-8). Further, 17 miRNA discriminated the HIV positive cases from the HIV negative ones (Supplementary Table 9). Conversely, none of the putative novel miRNA were differentially expressed in PBLs according to their relation with EMPC or BL. Of note, one of these novel putative miRNA mapped on the HIV genome. However, these preliminary observations need appropriate validation.

## DISCUSSION

The pathogenesis of PBL has not been clearly defined but most likely it involves deregulation of terminal B-cell differentiation and apoptosis, *via* the effect of *MYC* gene rearrangements and possibly EBV infection [3]. To better understand the viral program expressed in PBLs, we performed a complete assessment of EBV-encoded genes and proteins, demonstrating a non-canonical latency-associated program with the expression of latency II characterizing viral proteins along with sporadic lytic cycle reactivation. Nonetheless, the absence of *BLLF1*/gp350 expression in all the cases indicated an abortive lytic cycle in neoplastic cells. These results are consistent with previous findings, suggesting that EBV lytic reactivation may have pathogenic relevance in the context of both active and depressed immune response [6]. Intriguingly, we have recently demonstrated a non-canonical latency program with activation of the lytic cycle also in endemic BL cases, highlighting another overlap between PBL and BL [7].

The limited knowledge on PBL biology likely contributes to suboptimal management and poor prognosis of the patients. Therefore, in the present study we attempted to shed new light on the molecular bases of PBL. We studied for the first time the miRNA expression profile of PBL and compared it with those of BL and EMPC, chosen as the closest neoplastic counterparts considering morphological, immunophenotypic, genetic and epidemiological factors.

For the first time we observed that PBL include at least two molecular subgroups that presented with clinico-pathological peculiar features, including *MYC* rearrangements and HIV-association that appeared as main drivers of this sub-classification. On the other hand, this might be also due to a dissimilar histogenesis, genetic background and/or microenvironmental influences [5, 7, 8]. Interestingly, we found that the two PBL subgroups differed for the expression of proteins, the regulation of which might be sustained by the differentially expressed miRNA. These proteins turned out to be involved in relevant cellular processes including cell cycle and proliferation, signal transduction, apoptosis, viral replication, cell metabolism, epigenetic regulation of transcription (*via EZH2*) and differentiation. Of note, our findings unveiled another overlap between PBL and BL related to the metabolic reprogramming of the neoplastic cells [9]. In fact, both PBL and BL showed an activation of the lipid metabolism characterized by the expression of adipophilin, likely related to MYC activity as previously reported in the B-cell setting [10, 11].

Furthermore, we identified specific differences between PBL and both BL and EMPC as far as miRNA expression was concerned. Of note, based on the expression of as few as 4 miRNA, we could efficiently perform a molecular differential diagnosis between PBL and EMPC, even when anaplastic/plasmablastic EMPC were included. The latter, at present constitute real diagnostic challenges even for most experienced hematopathologists. Here, we demonstrated the efficiency of the molecular diagnostic tool in two independent cohorts of unselected cases, fulfilling the STARD criteria for molecular diagnostic. Phase 4 diagnostic accuracy studies are now warranted to prospectively evaluate the clinical utility of the test.

Finally, we observed that PBL molecular features are at least in part dependent on HIV status. Different mechanisms might be in place how HIV might contribute to PBL pathogenesis: duration and the degree of immunodeficiency, loss of immune control of oncogenic herpesviruses as EBV, chronic B-cell proliferation/exhaustion due to chronic antigenic stimulation, and/or factors unrelated to immune dysfunction, including a theoretically possible direct oncogenic effect. Actually, the involvement of HIV infection in PBL pathogenesis may explain the high frequency of this virus in PBL patients. In our analysis, HIV-positive and HIV-negative PBL cases differed in terms of cellular miRNA. This might be due in part to HIV itself by its own miRNA. However, only when qPCR was performed we could detect very low amounts of HIV-encoded miRNA in PBL cases. This likely reflected the inferior sensitivity of RNAseq analysis, especially when filters improving specificity and robustness are applied to RNAseq analysis. Indeed, the detection of viral miRNA is more likely consistent with the hypothesis that they were encoded in scattered T-cells, macrophages or dendritic cells rather than in the neoplastic clone. Nevertheless, their potential pathogenetic role in PBL cannot be excluded as miRNA are easily transferred through the tissues in the tumor microenvironment. Moreover, we identified by NGS a putative novel miRNA mapping on the HIV genome and highly expressed by PBL cells, the significance of which definitely need further analysis.

The major limitation of the study is probably the relatively low number of PBL cases analyzed (N=23). Nonetheless, it should be noted that all results have been validated in a cohort of cases independent from the discovery one, this making quite solid our assumptions [12].

In conclusion, the present study offers novel insights on PBL biology and pathogenesis that might be useful for the future management of this orphan disease in terms of classification, diagnosis and treatment. Particularly, an improved understanding of the complex interplay between genetic lesion and viral effects, as well as the multiple mechanisms regulating EBV reactivation might lead to the development of more specific therapies including miRNA-targeted therapies, MYC signaling pathways inhibitors, and combination of EBV lytic phase induction and anti-EBV drugs [13].

## MATERIALS AND METHODS

### Cases selection, immunohistochemistry, fluorescent *in situ* hybridization (FISH) and EBER-in-situ hybridization

For this study 23 PBL, 17 EMPC and 18 BL cases (9 endemic, 9 sporadic), collected at the Departments of Pathology of Aga Khan University Hospital, Nairobi (Kenya), Aga Khan Hospital, Kisumu (Kenya), Terni Hospital (Italy), and Siena University Hospital (Italy), were studied. Thirteen PBL, 7 EMPC and 9 BL (5 endemic) cases were used as discovery cohort for NGS-based miRNA profiling and EBV-latency assessment; 10 PBL, 10 EMPC and 10 BL cases were used as independent validation set of NGS analyses and were studied by qPCR and immunohistochemistry. The cases were reviewed by expert hemato-pathologists and diagnoses were confirmed by morphology on histological slides stained with haematoxylin and eosin (H&E) and Giemsa, and by immunophenotyping, according to the WHO [1, 14]. The following clinical data were collected: age, sex, disease location at presentation and an associated context of immunodeficiency.

Immunohistochemical studies were performed on representative paraffin sections from each case as previously reported [7, 15]. A large panel of antibodies (CD20, PAX-5, CD38, CD138, CD79a, CD10, BCL6, BCL2, MUM1, CD56, Igk, Igl) was applied to assess plasma cell differentiation. MYC protein expression and the proliferative index (by Ki-67) was evaluated. To rule out a diagnosis of ALK-positive large B-cell lymphoma or diffuse large B-cell lymphoma arising in multicentric Castlemann disease, immunohistochemical evaluation of ALK protein and HHV-8 was checked as well.

We studied the latency type of EBV by assessing the expression of some latent (EBNA-1, EBNA-2, EBNA-3, LMP-1, LMP-2A) and lytic proteins (EA-D, EA-R, BZLF1/ZEBRA, BLLF1/gp350) by immunohistochemistry. *In situ* hybridization for the non-coding EBERs was carried out in each sample as previously described [7]. A control slide, prepared from a paraffin-embedded tissue block containing metastatic nasopharyngeal carcinoma in a lymph node, accompanied each hybridization run.

In addition, the expression level of the proteins (adipophilin, PPARGC1A, FOXK2, PBX2, and PTEN) encoded by the genes found to be potentially targeted by the cellular and viral miRNA deregulated in the studied tumors was checked in the different primary tumors to highlight their positive or negative regulation by miRNA [16]. Differences in protein expression between cases were analyzed using the Chi-square test with p<0.05 being statistical significant.

For all the stains, negative controls were obtained for each case by replacing the specific antibody with non-immune serum immunoglobulins at the same concentration as the primary antibody. Sections of reactive lymphoid tissue and of lymph node with infectious mononucleosis were used as positive control for plasma cell differentiation and EBV latency type and lytic phase.

FISH analysis for *MYC* rearrangements was performed using both dual-fusion probes and split-signal probes for IGH and IGL *loci* as well as an LSI IGH/*MYC* CEP 8 Tri-color dual-fusion probe (Vysis, Abbott Molecular IL, USA) [15].

All the clinical, morphological, immunophenotypical and cytogenetic features of the discovery cohort patients are summarized in Table 1.

### MicroRNA profiling

Total RNA was extracted from formalyn-fixed paraffed-embedded (FFPE) sections of primary tumors and reactive lymph nodes using the FFPE miRNA Easy kit (Qiagen, CA), according to the manufacturer's instructions. Small RNA libraries were prepared from 1 μg of an high-quality RNA (RNA Integrity Number≥8) with the TruSeq Small RNA kit (Illumina). 1×36 sequencing was performed on the Illumina MiSeq platform. 36-bp length raw sequences were demultiplexed using the Illumina pipeline CASAVA v1.8. A quality check of the run experiment was performed by FastQC (http://www.bioinformatics.babraham.ac.uk/projects/fastqc). Low quality reads and adpater sequences were trimmed off using Trimmomatic [17]. The high quality reads, with a length of 17–36-bp were clipped and subsequently aligned to the latest mirBase release (v 21 July 2014) [18] by Novoalign (http://www.novocraft.com) MiRNA expression profiles were built by calculating the sum of read counts for each miRNA, according to the alignment criteria. Variance-stabilizing transformed count data were used to build an Euclidean distance matrix, followed by hierarchical clustering analysis to study the intra-samples correlations. miRNA differential expression analysis was performed using Bioconductor's package DESeq [19]. The obtained read counts for each identified miRNA were first normalized by scaling for library size factors in order to deal with variation among samples. The differential expression values were estimated using a negative binomial distribution model and local regression to estimate the relationship between the dispersion and the mean of each miRNA. Raw values were considered as statistically significant for p <0.05. Following alignment and normalization, the data were analyzed using GeneSpring GX12 as previously described [20–25], by applying Pearson correlation (for unsupervised clustering) and two-tails Student t-test with p-value <0.05 and fold change >2 (for supervised clustering). The experimentally validated targets for the viral and cellular miRNA were searched in public databases. For the viral miRNA, we used VIRmiRNA (http://crdd.osdd.net/servers/virmirna/) [26]; for the cellular miRNA, miRWalk 2 (http://zmf.umm.uni-heidelberg.de/apps/zmf/mirwalk2/) [27, 28]. Only proved direct interaction were then considered. Pathway analysis was performed by Gene Set Enrichment Analysis (GSEA) [29].

### Reverse transcription-quantitative PCR (RT-qPCR)

The expression of EBV-encoded genes (*EBNA-1, EBNA-2, LMP-1, LMP-2, EBNA-3, BZLF-1 BMRF-1/Ea-D, BHRF-1/Ea-R,* BLLF1/*gp350*) characterizing the different latency programs, has been investigated by RT-qPCR using the QuantiTect SYBR Green PCR Kit (Qiagen, CA). The stably expressed housekeeping gene hypoxanthine-guanine phosphoribosyltransferase (HPRT) was used as an endogenous control and reference gene for relative quantification of each target gene. The relative expression is expressed as 2^ΔCt^, where ΔCt is defined as the difference in mean cycle thresholds of the gene of interest and HPRT.

The expression level of differentially expressed human (hsa-miR1304-3p, hsa-miR148a-3p, hsa-miR148a-5p, hsa-miR192-5p) and viral miRNA (EBV-miR-BART-10-5p, EBV-miR-BART-18-5p, EBV-miR-BART-19-3p), selected based on the score in differential analysis and the known biological relevance, was evaluated by using Taqman probes specific for each miRNA and for RNU6B (the endogenous control) (Apllied Viosystems, Applera, Italy). The relative expression of cellular miRNA was calculated using the 2^-∆∆ct^ formula; for viral miRNA the following formula was adopted: 2^-∆ct^ [30]. Primers used for qPCR analyses are detailed in Table 2.

### Diagnostic accuracy evaluation

A diagnostic accuracy analysis was designed to test the ability of a newly developed molecular classifier (in particular of the 4-miRNAs based classifier) to discriminate PBL and EPCM. Calculations of sensitivity, specificity, positive predictive value, negative predictive value, positive and negative likelihood ratio, and odd ratio were made by CATmaker software (Centre for Evidence Based Medicine, Oxford University, http://www.cebm.net). The study was conducted according to the STARD guidelines for biomarker testing (Supplementary Table 1).

## SUPPLEMENTARY MATERIALS FIGURES AND TABLES




















