# Supplementary material for: MicroRNAs sequencing unveils distinct molecular subgroups of plasmablastic lymphoma
Source: Oncotarget. 2017 Oct 31;8(64):107356–73. doi: 10.18632/oncotarget.22219 (PMC5746073; doi:10.18632/oncotarget.22219)
Supplement: Supplementary file 1 [file oncotarget-08-107356-s001.pdf]

MicroRNAs sequencing unveils distinct molecular subgroups of plasmablastic lymphoma

SUPPLEMENTARY MATERIALS

For Supplementary Tables see in Supplementary Files

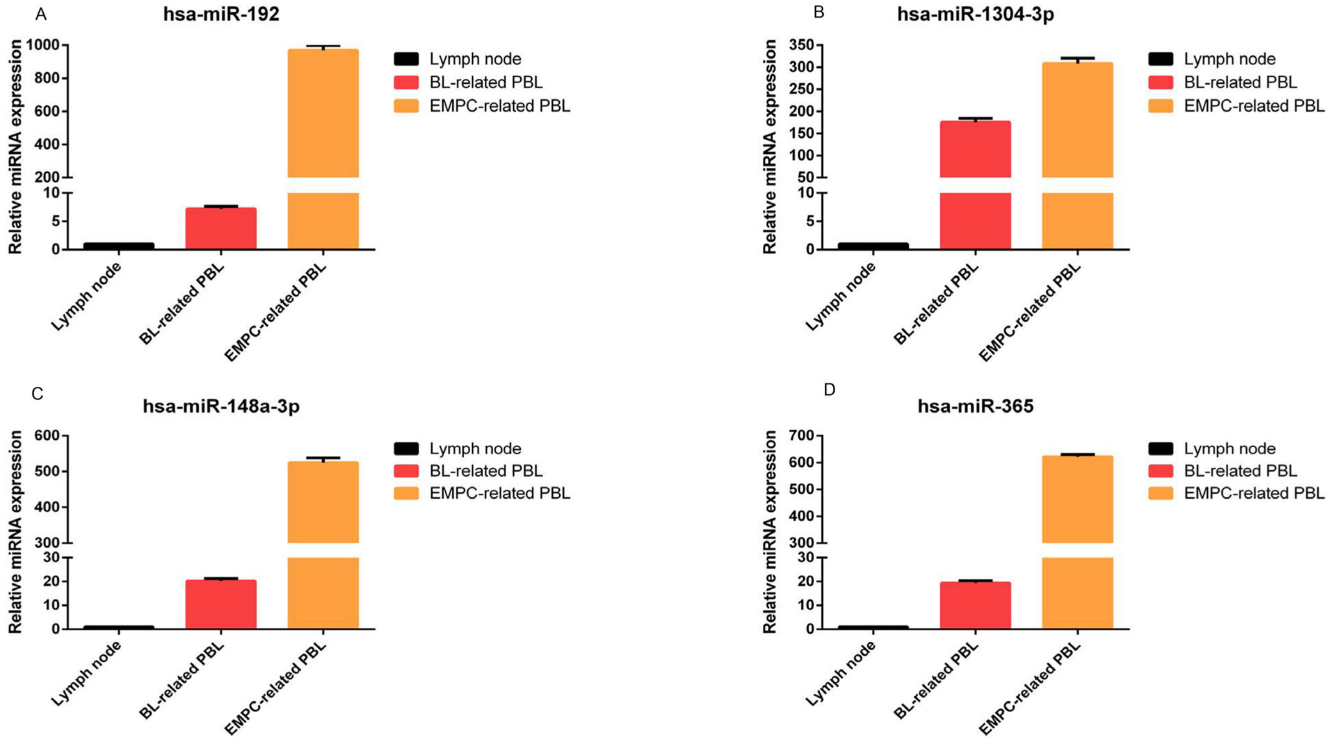

Supplementary Figure 1: Differential expression of hsa-miR-192-5p (**A**), hsa-miR-1304-3p (**B**), hsa-miR-148a-3p (**C**) and hsa-miR-365-3p (**D**) expression in BL-related versus EMPC-related PBLs validation group**.** Values are expressed as mean ± standard deviation (ST). Statistical significance was determined with Student's unpaired t-test ($p < 0.005$).

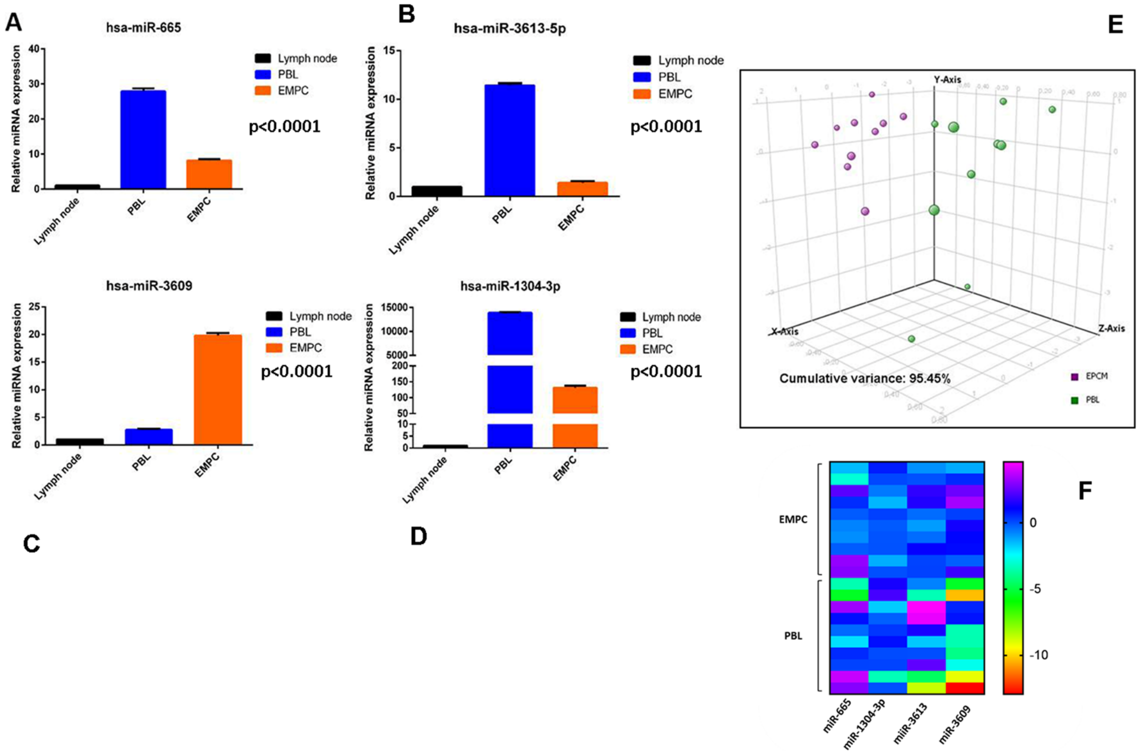

Supplementary Figure 2: hsa-miR-3613-5p, hsa-miR-1304-3p and hsa-miR-3609 expression in PBL versus EMPC validation cohort by using RT-qPCR (A). Values are expressed as mean ± standard deviation (ST). Statistical significance was determined with Student's unpaired t-test ($p < 0.005$). Principal component analysis indicated that based on the expression of as few as 4 miRNAs (**A**-**D**), an independent set of case was correctly classified as PBL or EPCM, the two entities being clearly distinct (**E**). Based on the expression of such miRNA, cases were then clustered (**F**)

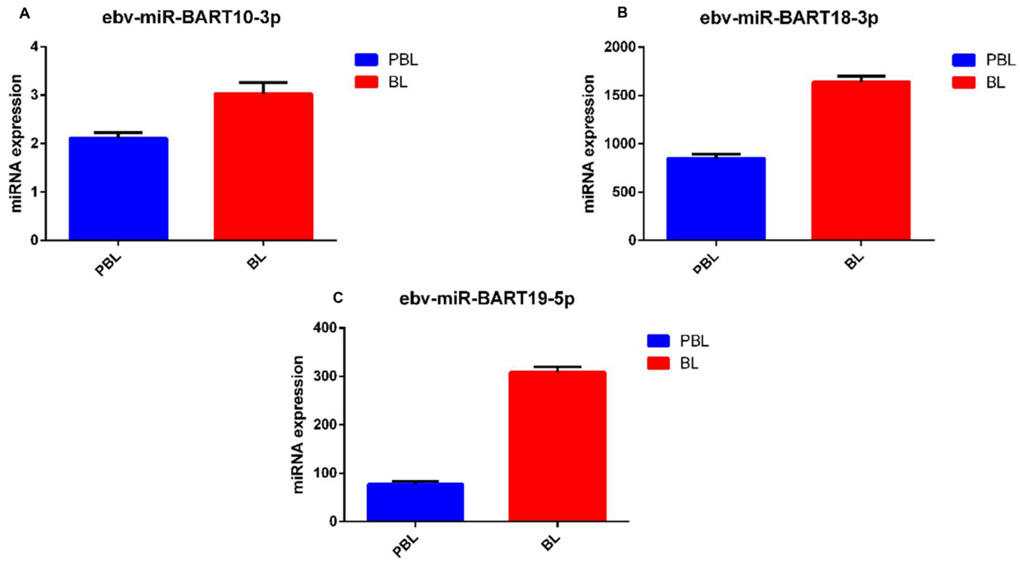

Supplementary Figure 3: EBV-viral miRNAs (EBV-miR-Bart10-3p (**A**), EBV-miR-18-3p (**B**), EBV-miR-19-5p (**C**)) expression in PBL versus BL validation sets by using RT-qPCR**.** Values are expressed as mean ± standard deviation (ST). Statistical significance was determined with Student's unpaired t-test ($p < 0.005$)..
