# Supplementary material for: MicroRNAs sequencing unveils distinct molecular subgroups of plasmablastic lymphoma
Source: Oncotarget. 2017 Oct 31;8(64):107356–73. doi: 10.18632/oncotarget.22219 (PMC5746073; doi:10.18632/oncotarget.22219)
Supplement: Supplementary file 2 [file oncotarget-08-107356-s002.docx]

**Supplementary Table 1. STARD guidelines for biomarker testing.**

|  | **Item** |  | **On page #** |
| --- | --- | --- | --- |
|  | **#** |  |  |
| TITLE/ABSTRACT/ | 1 | Identify the article as a study of diagnostic accuracy (recommend | / |
| KEYWORDS |  | MeSH heading ’sensitivity and specificity’). |  |
|  |  |  |  |
|  |  |  |  |
| INTRODUCTION | 2 | State the research questions or study aims, such as estimating | See Introduction |
|  |  | diagnostic accuracy or comparing accuracy between tests or across |  |
|  |  | participant groups. |  |
|  |  |  |  |
|  |  |  |  |
| METHODS |  |  |  |
|  |  |  |  |
| *Participants* | 3 | Describe the study population: The inclusion and exclusion criteria, | See Methods |
|  |  | setting and locations where the data were collected. |  |
|  |  |  |  |
|  | 4 | Describe participant recruitment: Was recruitment based on presenting | Consecutive patients who received the |
|  |  | symptoms, results from previous tests, or the fact that the participants | index test |
|  |  | had received the index tests or the reference standard? |  |
|  |  |  |  |
|  | 5 | Describe participant sampling: Was the study population a consecutive | Consecutive cases |
|  |  | series of participants defined by the selection criteria in items 3 and 4? |  |
|  |  | If not, specify how participants were further selected. |  |
|  |  |  |  |
|  | 6 | Describe data collection: Was data collection planned before the index | Restrospective study |
|  |  | test and reference standard were performed (prospective study) or after |  |
|  |  | (retrospective study)? |  |
|  |  |  |  |
| *Test methods* | 7 | Describe the reference standard and its rationale. | See methods |
|  |  |  |  |
|  | 8 | Describe technical specifications of material and methods involved | See methods |
|  |  | including how and when measurements were taken, and/or cite |  |
|  |  | references for index tests and reference standard. |  |
|  |  |  |  |
|  | 9 | Describe definition of and rationale for the units, cutoffs and/or | Methods |
|  |  | categories of the results of the index tests and the reference standard. |  |
|  |  |  |  |
|  | 10 | Describe the number, training and expertise of the persons executing | Expert in hematopathology and |
|  |  | and reading the index tests and the reference standard. | molecular pathology, respectively |
|  |  |  |  |
|  | 11 | Describe whether or not the readers of the index tests and reference | Blinded in the validation set (not possible |
|  |  | standard were blind (masked) to the results of the other test and | in the training set) |
|  |  | describe any other clinical information available to the readers. |  |
|  |  |  |  |
| *Statistical methods* | 12 | Describe methods for calculating or comparing measures of diagnostic | Catmaker (see methods) |
|  |  | accuracy, and the statistical methods used to quantify uncertainty (e.g. |  |
|  |  | 95% confidence intervals). |  |
|  |  |  |  |
|  | 13 | Describe methods for calculating test reproducibility, if done. | / |
|  |  |  |  |
|  |  |  |  |
| RESULTS |  |  |  |
|  |  |  |  |
| *Participants* | 14 | Report when study was done, including beginning and ending dates of | See methods |
|  |  | recruitment. |  |
|  |  |  |  |

|  | 15 | Report clinical and demographic characteristics of the study population | See Methods and Tables |
| --- | --- | --- | --- |
|  |  | (e.g. age, sex, spectrum of presenting symptoms, comorbidity, current |  |
|  |  | treatments, recruitment centers). |  |
|  |  |  |  |
|  | 16 | Report the number of participants satisfying the criteria for inclusion | All cases initially included were studied |
|  |  | that did or did not undergo the index tests and/or the reference |  |
|  |  | standard; describe why participants failed to receive either test (a flow |  |
|  |  | diagram is strongly recommended). |  |
|  |  |  |  |
| *Test results* | 17 | Report time interval from the index tests to the reference standard, and | No treatment. Interval of around 12 |
|  |  | any treatment administered between. | months (from diagnosis revision for |
|  |  |  | study inclusion to validation set analysis) |
|  |  |  |  |
|  | 18 | Report distribution of severity of disease (define criteria) in those with | / |
|  |  | the target condition; other diagnoses in participants without the target |  |
|  |  | condition. |  |
|  |  |  |  |
|  | 19 | Report a cross tabulation of the results of the index tests (including | See tables |
|  |  | indeterminate and missing results) by the results of the reference |  |
|  |  | standard; for continuous results, the distribution of the test results by |  |
|  |  | the results of the reference standard. |  |
|  |  |  |  |
|  | 20 | Report any adverse events from performing the index tests or the | / |
|  |  | reference standard. |  |
|  |  |  |  |
| *Estimates* | 21 | Report estimates of diagnostic accuracy and measures of statistical | See Tables and Figures |
|  |  | uncertainty (e.g. 95% confidence intervals). |  |
|  |  |  |  |
|  | 22 | Report how indeterminate results, missing responses and outliers of the | See Figures |
|  |  | index tests were handled. |  |
|  |  |  |  |
|  | 23 | Report estimates of variability of diagnostic accuracy between | / |
|  |  | subgroups of participants, readers or centers, if done. |  |
|  |  |  |  |
|  | 24 | Report estimates of test reproducibility, if done. | / |
|  |  |  |  |
|  |  |  |  |
| DISCUSSION | 25 | Discuss the clinical applicability of the study findings. | See Discussion |
|  |  |  |  |
