# Supplementary material for: MicroRNAs sequencing unveils distinct molecular subgroups of plasmablastic lymphoma
Source: Oncotarget. 2017 Oct 31;8(64):107356–73. doi: 10.18632/oncotarget.22219 (PMC5746073; doi:10.18632/oncotarget.22219)
Supplement: Supplementary file 3 [file oncotarget-08-107356-s003.docx]

**Supplementary Table 2.** List of the 48 differentially expressed microRNAs (10 cellular and 38 EBV-encoded) between plasmablastic lymphoma and extramedullary plasmacytoma.

| **miRNA** | **p** | **FC (abs)** | **Regulation in PBL** |
| --- | --- | --- | --- |
|  |  | |  |
| ebv-miR-BART10-3p | 0.014494695 | 3,68E+07 | up |
| ebv-miR-BART10-5p | 0.004778709 | 2,9048457 | up |
| ebv-miR-BART11-3p | 0.021626908 | 23258.896 | up |
| ebv-miR-BART11-5p | 0.012132136 | 374.66306 | up |
| ebv-miR-BART12 | 0.017350873 | 42.095806 | up |
| ebv-miR-BART13-3p | 0.023338085 | 64 | up |
| ebv-miR-BART13-5p | 0.013841942 | 270.02017 | up |
| ebv-miR-BART1-3p | 0.01457786 | 64 | up |
| ebv-miR-BART14-3p | 0.031335708 | 398.2036 | up |
| ebv-miR-BART14-5p | 0.03719868 | 2.7750762 | up |
| ebv-miR-BART15 | 0.025378168 | 4.219064 | up |
| ebv-miR-BART1-5p | 0.024425354 | 274.16504 | up |
| ebv-miR-BART16 | 0.04266382 | 436694.03 | up |
| ebv-miR-BART17-3p | 0.012719589 | 569.61597 | up |
| ebv-miR-BART17-5p | 0.019113962 | 5994.609 | up |
| ebv-miR-BART18-3p | 0.005118324 | 1277.1221 | up |
| ebv-miR-BART18-5p | 0.012309627 | 9.90181 | up |
| ebv-miR-BART19-3p | 0.004510972 | 9183.542 | up |
| ebv-miR-BART19-5p | 0.012688287 | 380.41446 | up |
| ebv-miR-BART20-3p | 0.012007042 | 2.346921 | up |
| ebv-miR-BART21-3p | 0.025480364 | 9.90181 | up |
| ebv-miR-BART22 | 0.02996077 | 1971.4686 | up |
| ebv-miR-BART2-3p | 0.048129003 | 3.8213065 | up |
| ebv-miR-BART2-5p | 0.020195836 | 2613.2842 | up |
| ebv-miR-BART3-3p | 0.027982349 | 80.43048 | up |
| ebv-miR-BART3-5p | 0.016911246 | 22.032148 | up |
| ebv-miR-BART4-3p | 0.038767755 | 14.381615 | up |
| ebv-miR-BART4-5p | 0.019079057 | 26.250734 | up |
| ebv-miR-BART5-3p | 0.01564096 | 2.2250628 | up |
| ebv-miR-BART5-5p | 0.023106847 | 34826.832 | up |
| ebv-miR-BART6-3p | 0.009791852 | 16013.859 | up |
| ebv-miR-BART6-5p | 0.021794938 | 460.21182 | up |
| ebv-miR-BART7-3p | 0.03267353 | 1.35E+07 | up |
| ebv-miR-BART7-5p | 0.007673319 | 142.40404 | up |
| ebv-miR-BART8-3p | 0.029387834 | 3517.218 | up |
| ebv-miR-BART8-5p | 0.021917798 | 3766.7925 | up |
| ebv-miR-BART9-3p | 0.034293603 | 2673.6873 | up |
| ebv-miR-BART9-5p | 0.01330993 | 1147.9427 | up |
| hsa-miR-1275 | 0.039673198 | 8.63317 | up |
| hsa-miR-1304-3p | 0.04551583 | 3.1587212 | up |
| hsa-miR-218-5p | 0.009204442 | 2.7962947 | down |
| hsa-miR-221-3p | 0.04577592 | 305.01736 | down |
| hsa-miR-3609 | 9.26E-04 | 10.52395 | down |
| hsa-miR-3613-5p | 0.021165587 | 2.3829477 | up |
| hsa-miR-375 | 0.024927238 | 25.853855 | down |
| hsa-miR-3922-5p | 0.045415826 | 2.0152922 | up |
| hsa-miR-625-3p | 0.04925378 | 3.4347847 | up |
| hsa-miR-665 | 0.011589593 | 4.416358 | up |
