# Supplementary material for: MicroRNAs sequencing unveils distinct molecular subgroups of plasmablastic lymphoma
Source: Oncotarget. 2017 Oct 31;8(64):107356–73. doi: 10.18632/oncotarget.22219 (PMC5746073; doi:10.18632/oncotarget.22219)
Supplement: Supplementary file 4 [file oncotarget-08-107356-s004.docx]

**Supplementary Table 3.** List of the 42 differentially expressed microRNAs (23 cellular and 19 EBV miRNA) between plasmablastic lymphoma and Burkitt lymphoma.

| **miRNA** | **p** | **FC (abs)** | **Regulation in PBL** |
| --- | --- | --- | --- |
| ebv-miR-BART10-5p | 0.006791761 | 2.4901617 | down |
| ebv-miR-BART11-3p | 0.037498765 | 3174.8633 | down |
| ebv-miR-BART12 | 0.03965184 | 17.077408 | down |
| ebv-miR-BART13-5p | 0.030023802 | 72.909485 | down |
| ebv-miR-BART14-5p | 0.039804325 | 2.431845 | down |
| ebv-miR-BART17-3p | 0.025096882 | 153.80482 | down |
| ebv-miR-BART17-5p | 0.026609913 | 1418.4358 | down |
| ebv-miR-BART18-3p | 0.041157667 | 111.03505 | down |
| ebv-miR-BART18-5p | 0.008088547 | 8.488267 | down |
| ebv-miR-BART19-3p | 0.019128906 | 931.39343 | down |
| ebv-miR-BART19-5p | 0.01707289 | 149.31573 | down |
| ebv-miR-BART20-3p | 0.021549927 | 2.011884 | down |
| ebv-miR-BART21-3p | 0.03472866 | 6.737142 | down |
| ebv-miR-BART2-3p | 0.045557205 | 3.3486736 | down |
| ebv-miR-BART2-5p | 0.037767462 | 485.4156 | down |
| ebv-miR-BART4-5p | 0.033187144 | 13.715902 | down |
| ebv-miR-BART7-3p | 0.04277328 | 888298.44 | down |
| ebv-miR-BART7-5p | 0.014932586 | 52.324043 | down |
| ebv-miR-BART9-5p | 0.03752318 | 171.11278 | down |
| hsa-miR-1246 | 0.011286354 | 1.33E+14 | up |
| hsa-miR-1273f | 6.02E-04 | 2.7703831 | up |
| hsa-miR-1469 | 0.047018066 | 16.976536 | up |
| hsa-miR-148a-5p | 0.01317542 | 7.812649 | down |
| hsa-miR-3141 | 0.04192238 | 6.48E+16 | up |
| hsa-miR-3609 | 0.03464726 | 2.4901617 | up |
| hsa-miR-3648 | 0.039415363 | 5.253075 | up |
| hsa-miR-425-5p | 0.01428534 | 9.960645 | up |
| hsa-miR-4417 | 0.03250875 | 8.84768 | up |
| hsa-miR-4466 | 0.028467217 | 5.0997524 | up |
| hsa-miR-4488 | 0.017206004 | 5984591 | up |
| hsa-miR-4492 | 0.007730778 | 1.24E+09 | up |
| hsa-miR-4521 | 0.008676537 | 2.134676 | up |
| hsa-miR-4532 | 0.025652096 | 2.68E+07 | up |
| hsa-miR-4539 | 0.028557671 | 2.134676 | up |
| hsa-miR-4787-5p | 0.026852762 | 7.495283 | up |
| hsa-miR-4791 | 0.003031531 | 770.5489 | up |
| hsa-miR-494-3p | 0.024801403 | 44590.375 | up |
| hsa-miR-6510-5p | 0.04812371 | 128 | up |
| hsa-miR-6743-5p | 0.034500074 | 7.406997 | up |
| hsa-miR-6803-5p | 0.022911137 | 3.0278432 | up |
| hsa-miR-6869-5p | 0.017606273 | 3595.4731 | up |
| hsa-miR-7111-3p | 0.029701255 | 2.2515838 | up |
