# Supplementary material for: MicroRNAs sequencing unveils distinct molecular subgroups of plasmablastic lymphoma
Source: Oncotarget. 2017 Oct 31;8(64):107356–73. doi: 10.18632/oncotarget.22219 (PMC5746073; doi:10.18632/oncotarget.22219)
Supplement: Supplementary file 5 [file oncotarget-08-107356-s005.docx]

**Supplementary Table 4.** List of the 68 differentially expressed microRNAs between HIV-positive and HIV-negative plasmablastic lymphoma.

| **miRNA** | **p** | **FC (abs)** | **Regulation** |
| --- | --- | --- | --- |
| hsa-let-7b-3p | 2.57E-04 | 2.4199882 | up |
| hsa-let-7b-5p | 0.010559293 | 5404.703 | up |
| hsa-let-7d-5p | 0.01646335 | 3.54307 | up |
| hsa-miR-100-5p | 0.04331178 | 5.9587092 | up |
| hsa-miR-106b-3p | 0.010191316 | 5.278032 | up |
| hsa-miR-106b-5p | 0.04619508 | 9.513657 | up |
| hsa-miR-10b-5p | 0.023008613 | 3.30E+07 | up |
| hsa-miR-125a-5p | 0.01648309 | 38.054626 | up |
| hsa-miR-125b-1-3p | 0.043265637 | 2.0349593 | up |
| hsa-miR-126-3p | 0.001710106 | 251.60214 | up |
| hsa-miR-127-3p | 0.033425618 | 67.4151 | up |
| hsa-miR-132-3p | 0.023671202 | 2.979355 | up |
| hsa-miR-134-5p | 0.042120945 | 5.0982423 | up |
| hsa-miR-139-5p | 0.003478305 | 2.5491214 | up |
| hsa-miR-140-3p | 0.040174495 | 115.36009 | up |
| hsa-miR-143-3p | 0.037074823 | 15024.194 | up |
| hsa-miR-145-5p | 0.007839669 | 31.450256 | up |
| hsa-miR-146a-5p | 0.01278072 | 59.714096 | up |
| hsa-miR-151a-3p | 0.035840645 | 36.126865 | up |
| hsa-miR-151a-5p | 0.010817928 | 3.8637455 | up |
| hsa-miR-152-3p | 0.034645252 | 8.282119 | up |
| hsa-miR-155-5p | 0.02233786 | 97.00588 | up |
| hsa-miR-181a-3p | 0.008989137 | 2.3784142 | up |
| hsa-miR-181c-5p | 0.02719111 | 4.839977 | up |
| hsa-miR-193a-3p | 0.039865468 | 2.7798364 | up |
| hsa-miR-193b-3p | 0.031347785 | 59.714096 | up |
| hsa-miR-196b-5p | 0.036444213 | 2.4199882 | up |
| hsa-miR-197-3p | 0.012227654 | 13.928811 | up |
| hsa-miR-199a-3p | 0.011095375 | 12.337687 | up |
| hsa-miR-199a-5p | 0.022936901 | 71.01245 | up |
| hsa-miR-199b-5p | 0.023599725 | 9.350218 | up |
| hsa-miR-200c-3p | 0.028489472 | 4.594794 | up |
| hsa-miR-205-5p | 0.046048716 | 13777.247 | up |
| hsa-miR-214-3p | 0.001072369 | 7.08614 | up |
| hsa-miR-24-3p | 0.009809502 | 20.39297 | up |
| hsa-miR-27a-3p | 0.045108575 | 284.0497 | up |
| hsa-miR-27b-3p | 0.037576143 | 184.18346 | up |
| hsa-miR-27b-5p | 0.02153633 | 2.106722 | up |
| hsa-miR-28-3p | 0.002059643 | 48.50294 | up |
| hsa-miR-29c-5p | 0.024921691 | 2.5936792 | up |
| hsa-miR-30a-5p | 0.030055713 | 53.81737 | up |
| hsa-miR-30e-3p | 0.046484742 | 3.7321322 | up |
| hsa-miR-31-5p | 0.03115625 | 5.278032 | up |
| hsa-miR-3180-3p | 0.005529448 | 5.278032 | up |
| hsa-miR-324-3p | 7.28E-06 | 4.594794 | up |
| hsa-miR-324-5p | 0.024743747 | 2.4622889 | up |
| hsa-miR-339-5p | 0.045017295 | 3.1383367 | up |
| hsa-miR-342-3p | 0.029932043 | 50.213367 | up |
| hsa-miR-365a-3p | 0.002382816 | 2.685145 | up |
| hsa-miR-365b-3p | 0.01950378 | 2.3375545 | up |
| hsa-miR-377-5p | 2.31E-29 | 2 | up |
| hsa-miR-378d | 0.024921691 | 2.5936792 | up |
| hsa-miR-409-3p | 0.043110028 | 2.979355 | up |
| hsa-miR-423-3p | 0.036647916 | 42.224243 | up |
| hsa-miR-424-5p | 0.008075885 | 10.556064 | up |
| hsa-miR-450a-5p | 2.86E-04 | 3.1931934 | up |
| hsa-miR-452-5p | 0.001539338 | 3.031433 | up |
| hsa-miR-500a-3p | 0.024182294 | 3.0314329 | up |
| hsa-miR-574-3p | 0.020066956 | 3.7973683 | up |
| hsa-miR-574-5p | 2.78E-04 | 3.931282 | up |
| hsa-miR-625-3p | 0.020983625 | 6.6116023 | up |
| hsa-miR-708-5p | 7.62E-06 | 4.438278 | up |
| hsa-miR-744-5p | 0.012200036 | 3.1383367 | up |
| hsa-miR-769-5p | 0.001492048 | 6.1688433 | up |
| hsa-miR-874-3p | 0.010191316 | 5.278032 | up |
| hsa-miR-92b-3p | 0.03764931 | 355.81915 | up |
| hsa-miR-98-5p | 0.008844344 | 4 | up |
| hsa-miR-99b-3p | 0.003990301 | 2.979355 | up |
