# Supplementary material for: MicroRNAs sequencing unveils distinct molecular subgroups of plasmablastic lymphoma
Source: Oncotarget. 2017 Oct 31;8(64):107356–73. doi: 10.18632/oncotarget.22219 (PMC5746073; doi:10.18632/oncotarget.22219)
Supplement: Supplementary file 6 [file oncotarget-08-107356-s006.docx]

**Supplementary Table 5.** Analysis of genes targeted by the dysregulated microRNAs in plasmablastic lymphoma using GSEA.

| **Gene Set Name** | **# Genes in Gene Set (K)** | **Description** | **# Genes in Overlap (k)** | **k/K** | **p-value** | **FDR q-value** |
| --- | --- | --- | --- | --- | --- | --- |
| BENPORATH_MYC_MAX_TARGETS | 775 | Set 'Myc targets2': targets of c-Myc [GeneID=4609] and Max [GeneID=4149] identified by ChIP on chip in a Burkitt's lymphoma cell line; overlap set. | 129 | 0.167 | 3.53E-62 | 1.20E-59 |
| BENPORATH_NANOG_TARGETS | 988 | Set 'Nanog targets': genes upregulated and identified by ChIP on chip as Nanog [GeneID=79923] transcription factor targets in human embryonic stem cells. | 81 | 0.082 | 1.75E-34 | 9.23E-32 |
| BERENJENO_TRANSFORMED_BY_RHOA_UP | 536 | Genes up-regulated in NIH3T3 cells (fibroblasts) transformed by expression of contitutively active (Q63L) form of RHOA [GeneID=387] off plasmid vector. | 112 | 0.209 | 1.06E-64 | 4.37E-62 |
| BIDUS_METASTASIS_UP | 214 | Genes up-regulated in endometroid endometrial tumors from patients with lymph node metastases compared to those without the metastases. | 7 | 0.033 | 1.12E-07 | 0.0000719 |
| BIOPOLYMER_METABOLIC_PROCESS | 1684 | Genes annotated by the GO term GO:0043283. The chemical reactions and pathways involving biopolymers, long, repeating chains of monomers found in nature e.g. polysaccharides and proteins. | 232 | 0.138 | 6.04E-96 | 7.00E-93 |
| BLALOCK_ALZHEIMERS_DISEASE_DN | 1237 | Genes down-regulated in brain from patients with Alzheimer's disease. | 129 | 0.104 | 4.44E-44 | 1.51E-41 |
| BLALOCK_ALZHEIMERS_DISEASE_UP | 1691 | Genes up-regulated in brain from patients with Alzheimer's disease. | 210 | 0.124 | 5.56E-78 | 3.58E-75 |
| CAIRO_HEPATOBLASTOMA_CLASSES_UP | 605 | Genes up-regulated in robust Cluster 2 (rC2) of hepatoblastoma samples compared to those in the robust Cluster 1 (rC1). | 138 | 0.228 | 3.83E-85 | 3.16E-82 |
| DACOSTA_UV_RESPONSE_VIA_ERCC3_COMMON_DN | 483 | Common down-regulated transcripts in fibroblasts expressing either XP/CS or TDD mutant forms of ERCC3 [GeneID=2071], after UVC irradiation. | 55 | 0.114 | 9.88E-31 | 3.58E-28 |
| DACOSTA_UV_RESPONSE_VIA_ERCC3_DN | 855 | Genes down-regulated in fibroblasts expressing mutant forms of ERCC3 [GeneID=2071] after UV irradiation. | 144 | 0.168 | 3.49E-70 | 1.69E-67 |
| DANG_BOUND_BY_MYC | 1103 | Genes whose promoters are bound by MYC [GeneID=4609], according to MYC Target Gene Database. | 193 | 0.175 | 8.18E-98 | 1.18E-94 |
| DIAZ_CHRONIC_ MEYLOGENOUS_ LEUKEMIA_UP | 1382 | Genes up-regulated in CD34+ [GeneID=947] cells isolated from bone marrow of CML (chronic myelogenous leukemia) patients, compared to those from normal donors. | 216 | 0.156 | 6.81E-100 | 1.31E-96 |
| DODD_NASOPHARYNGEAL_CARCINOMA_DN | 1375 | Genes down-regulated in nasopharyngeal carcinoma (NPC) compared to the normal tissue. | 191 | 0.139 | 6.74E-79 | 4.88E-76 |
| FLECHNER_BIOPSY_KIDNEY_TRANSPLANT_OK_VS_DONOR_UP | 555 | Genes up-regulated in kidney biopsies from patients with well functioning kidneys more than 1-year post transplant compared to the biopsies from normal living kidney donors. | 90 | 0.162 | 2.86E-46 | 1.18E-43 |
| GARY_CD5_TARGETS_UP | 473 | Genes up-regulated in Daudi cells (B lymphocytes) stably expressing CD5 [GeneID=921] off a plasmid vector. | 8 | 0.017 | 1.98E-06 | 0.000582 |
| GRAESSMANN_APOPTOSIS_BY_DOXORUBICIN_DN | 1781 | Genes down-regulated in ME-A cells (breast cancer) undergoing apoptosis in response to doxorubicin [PubChem=31703]. | 238 | 0.134 | 1.00E-95 | 6.67E-93 |
| GROSS_HYPOXIA_VIA_ELK3_UP | 209 | Genes up-regulated in SEND cells (skin endothelium) at hypoxia with ELK3 [GeneID=2004] knockdown by RNAi. | 6 | 0.029 | 2.01E-06 | 0.000582 |
| JOHNSTONE_PARVB_TARGETS_3_DN | 918 | Genes down-regulated upon overexpression of PARVB [GeneID=29780] in MDA-MB-231 cells (breast cancer) cultured in 3D Matrigel only. | 84 | 0.092 | 2.66E-39 | 1.93E-36 |
| KEGG_PATHWAYS_IN_CANCER | 328 | Pathways in cancer | 7 | 0.021 | 1.96E-06 | 0.000582 |
| KIM_ALL_DISORDERS_OLIGODENDROCYTE_NUMBER_CORR_UP | 756 | Genes whose expression was significantly and positively correlated with the number of perineuronal oligodendrocytes in the layer III of BA9 brain region. | 10 | 0.013 | 8.98E-07 | 0.0004 |
| KINSEY_TARGETS_OF_EWSR1_FLII_FUSION_UP | 1278 | Genes up-regulated in TC71 and EWS502 cells (Ewing's sarcoma) by EWSR1-FLI1 [GeneID=2130;2314] as inferred from RNAi knockdown of this fusion protein | 175 | 0.137 | 4.23E-71 | 2.23E-68 |
| KOINUMA_TARGETS_OF_SMAD2_OR_SMAD3 | 824 | Genes with promoters occupied by SMAD2 or SMAD3 [GeneID=4087, 4088] in HaCaT cells (keratinocyte) according to a ChIP-chip analysis. | 106 | 0.129 | 1.34E-44 | 4.84E-42 |
| KRIGE_RESPONSE_TO_TOSEDOSTAT_24HR_DN | 1011 | Genes down-regulated in HL-60 cells (acute promyelocytic leukemia, APL) after treatment with the aminopeptidase inhibitor tosedostat (CHR-2797) [PubChem=15547703] for 24 h. | 146 | 0.144 | 4.01E-62 | 1.29E-59 |
| LEE_BMP2_TARGETS_DN | 882 | Genes down-regulated in uterus upon knockout of BMP2 [GeneID=650]. | 154 | 0.175 | 1.94E-77 | 1.13E-74 |
| LOPEZ_MBD_TARGETS | 957 | Genes up-regulated in HeLa cells (cervical cancer) after simultaneus knockdown of all three MBD (methyl-CpG binding domain) proteins MeCP2,MBD1 and MBD2 [GeneID=4204;4152;8932] by RNAi. | 13 | 0.014 | 1.30E-08 | 0.0000125 |
| MARSON_BOUND_BY_E2F4_UNSTIMULATED | 728 | Genes with promoters bound by E2F4 [GeneID=1874] in unstimulated hybridoma cells. | 10 | 0.014 | 6.39E-07 | 0.000308 |
| MARSON_BOUND_BY_FOXP3_STIMULATED | 1022 | Genes with promoters bound by FOXP3 [GeneID=50943] in hybridoma cells stimulated by PMA [PubChem=4792] and ionomycin [PubChem=3733]. | 81 | 0.079 | 1.90E-33 | 9.17E-31 |
| MARSON_BOUND_BY_FOXP3_UNSTIMULATED | 1229 | Genes with promoters bound by FOXP3 [GeneID=50943] in unstimulated hybridoma cells. | 84 | 0.068 | 4.34E-30 | 1.40E-27 |
| NUCLEOBASENUCLEOSIDENUCLEOTIDE_AND_NUCLEIC_ACID_METABOLIC_PROCESS | 1244 | Genes annotated by the GO term GO:0006139. The chemical reactions and pathways involving nucleobases, nucleosides, nucleotides and nucleic acids. | 165 | 0.133 | 6.79E-65 | 3.02E-62 |
| NUYTTEN_EZH2_TARGETS_DN | 1024 | Genes down-regulated in PC3 cells (prostate cancer) after knockdown of EZH2 [GeneID=2146] by RNAi. | 11 | 0.011 | 1.86E-06 | 0.000582 |
| NUYTTEN_EZH2_TARGETS_UP | 1037 | Genes up-regulated in PC3 cells (prostate cancer) after knockdown of EZH2 [GeneID=2146] by RNAi. | 121 | 0.117 | 2.21E-46 | 9.33E-44 |
| NUYTTEN_NIPP1_TARGETS_DN | 848 | Genes down-regulated in PC3 cells (prostate cancer) after knockdown of NIPP1 [GeneID=5511] by RNAi. | 70 | 0.083 | 4.80E-30 | 1.46E-27 |
| NUYTTEN_NIPP1_TARGETS_UP | 769 | Genes up-regulated in PC3 cells (prostate cancer) after knockdown of NIPP1 [GeneID=5511] by RNAi. | 98 | 0.127 | 5.07E-59 | 9.79E-56 |
| PILON_KLF1_TARGETS_DN | 1972 | Genes down-regulated in erythroid progenitor cells from fetal livers of E13.5 embryos with KLF1 [GeneID=10661] knockout compared to those from the wild type embryos. | 262 | 0.313 | 1.91E-105 | 5.53E-102 |
| PILON_KLF1_TARGETS_DN | 1972 | Genes down-regulated in erythroid rogenitor cells from fetal livers of E13.5 embryos with KLF1 [GeneID=10661] knockout compared to those from the wild type embryos. | 230 | 0.117 | 5.10E-90 | 1.95E-86 |
| PROTEIN_METABOLIC_PROCESS | 1231 | Genes annotated by the GO term GO:0019538. The chemical reactions and pathways involving a specific protein, rather than of proteins in general. Includes protein modification. | 160 | 0.13 | 1.22E-61 | 3.73E-59 |
| PUJANA_ATM_PCC_NETWORK | 1442 | Genes constituting the ATM-PCC network of transcripts whose expression positively correlated (Pearson correlation coefficient, PCC>= 0.4) with that of ATM [GeneID=472] across a compendium of normal tissues. | 175 | 0.121 | 5.16E-63 | 1.87E-60 |
| PUJANA_BRCA1_ PCC_NETWORK | 1652 | Genes constituting the BRCA1-PCC network of transcripts whose expression positively correlated (Pearson correlation coefficient, PCC >= 0.4) with that of BRCA1 [GeneID=672] across a compendium of normal tissues. | 254 | 0.154 | 8.17E-117 | 4.73E-113 |
| PUJANA_CHEK2_PCC_NETWORK | 779 | Genes constituting the CHEK2-PCC network of transcripts whose expression positively correlates (Pearson correlation coefficient, PCC >= 0.4) with that of CHEK2 [GeneID=11200]. | 131 | 0.168 | 1.06E-63 | 4.11E-61 |
| REACTOME_CELL_CYCLE | 421 | Genes involved in Cell Cycle | 9 | 0.021 | 6.08E-08 | 0.000044 |
| REACTOME_CELL_CYCLE_MITOTIC | 325 | Genes involved in Cell Cycle, Mitotic | 9 | 0.028 | 6.61E-09 | 9.22E-06 |
| REGULATION_OF_CELLULAR_METABOLIC_PROCESS | 787 | Genes annotated by the GO term GO:0031323. Any process that modulates the frequency, rate or extent of the chemical reactions and pathways by which individual cells transform chemical substances. | 102 | 0.13 | 3.18E-43 | 9.69E-41 |
| REGULATION_OF_METABOLIC_PROCESS | 799 | Genes annotated by the GO term GO:0019222. Any process that modulates the frequency, rate or extent of the chemical reactions and pathways within a cell or an organism. | 103 | 0.129 | 1.98E-43 | 6.37E-41 |
| RNA_METABOLIC_PROCESS | 841 | Genes annotated by the GO term GO:0016070. The chemical reactions and pathways involving RNA, ribonucleic acid, one of the two main type of nucleic acid, consisting of a long, unbranched macromolecule formed from ribonucleotides joined in 3',5'-phosphodiester linkage. | 130 | 0.155 | 9.16E-59 | 2.65E-56 |
| RODRIGUES_THYROID_CARCINOMA_ANAPLASTIC_UP | 722 | Genes up-regulated in anaplastic thyroid carcinoma (ATC) compared to normal thyroid tissue. | 66 | 0.091 | 5.99E-31 | 2.31E-28 |
