# Supplementary material for: MicroRNAs sequencing unveils distinct molecular subgroups of plasmablastic lymphoma
Source: Oncotarget. 2017 Oct 31;8(64):107356–73. doi: 10.18632/oncotarget.22219 (PMC5746073; doi:10.18632/oncotarget.22219)
Supplement: Supplementary file 8 [file oncotarget-08-107356-s008.docx]

**Supplementary Table 7.** List of the novel 27 microRNAs differentially expressed between plasmablastic lymphoma and Burkitt lymphoma.

| **identifier** | **p** | **FC (abs)** | **Regulation in PBL** |
| --- | --- | --- | --- |
|  | |  |  |
| chr10_21746 | 0.004026761 | 1.3670877 | up |
| chr11_22039 | 0.014956713 | 1.2271734 | up |
| chr12_24445 | 0.011004179 | 1.30754 | up |
| chr13_25173 | 0.047554817 | 1.2646013 | up |
| chr15_27721 | 0.0476526 | 1.215823 | up |
| chr17_30135 | 0.008791829 | 1.3479561 | up |
| chr20_33361 | 0.004842241 | 1.3525517 | up |
| chr2_5475 | 4.81E-04 | 1.4029489 | up |
| chr2_5700 | 0.024816938 | 1.3103708 | up |
| chr3_7710 | 7.57E-04 | 1.3926086 | up |
| chr3_7980 | 0.021946356 | 1.2095509 | down |
| chr4_10349 | 0.018392576 | 1.3365637 | up |
| chr4_10501 | 0.01470893 | 1.2912712 | down |
| chr4_8900 | 0.005851438 | 1.3277054 | up |
| chr4_9433 | 0.037086077 | 1.5229769 | down |
| chr5_10788 | 0.03310244 | 1.244398 | up |
| chr5_11238 | 0.046082582 | 1.2117505 | up |
| chr5_12617 | 0.010490277 | 1.2874213 | up |
| chr6_13585 | 0.027175322 | 1.1599579 | down |
| chr7_14883 | 0.011350005 | 1.266333 | up |
| chr8_16748 | 0.010822408 | 1.3014464 | up |
| chr8_17517 | 0.031662878 | 1.2678071 | down |
| chr8_17886 | 0.004017088 | 1.4939424 | up |
| chr8_17933 | 0.010732843 | 1.3540013 | up |
| chrX_35230 | 0.004322581 | 1.4646341 | up |
| chrX_35382 | 0.048482686 | 1.1747084 | up |
| chrX_35618 | 0.004313959 | 1.3762636 | up |
