# Supplementary material for: MicroRNAs sequencing unveils distinct molecular subgroups of plasmablastic lymphoma
Source: Oncotarget. 2017 Oct 31;8(64):107356–73. doi: 10.18632/oncotarget.22219 (PMC5746073; doi:10.18632/oncotarget.22219)
Supplement: Supplementary file 9 [file oncotarget-08-107356-s009.docx]

**Supplementary Table 8.** List of the novel 7 microRNAs differentially expressed between plasmablastic lymphoma and extramedullary plasmacytoma.

| **identifier** | **p** | **FC (abs)** | **Regulation in PBL** |
| --- | --- | --- | --- |
|  |  |  |  |
| chr1_2919 | 0.0420128 | 1.274662 | down |
| chr2_5475 | 0.0367783 | 1.314439 | up |
| chr4_10349 | 0.0461787 | 1.36644 | up |
| chr5_12584 | 0.0379738 | 1.198282 | down |
| chr6_13585 | 0.0483802 | 1.146323 | down |
| chr8_17886 | 0.0420392 | 1.381068 | up |
| chr1_1755 | 0.0211932 | 1.098456 | up |
