# Supplementary material for: MicroRNAs sequencing unveils distinct molecular subgroups of plasmablastic lymphoma
Source: Oncotarget. 2017 Oct 31;8(64):107356–73. doi: 10.18632/oncotarget.22219 (PMC5746073; doi:10.18632/oncotarget.22219)
Supplement: Supplementary file 10 [file oncotarget-08-107356-s010.docx]

**Supplementary Table 9.** List of the novel 17 microRNAs discriminating the HIV-positive cases from the HIV-negative ones.

| **identifier** | **p** | **Regulation in HIV-** | **FC (abs)** |
| --- | --- | --- | --- |
|  |  |  |  |
| chr1_1034 | 0.010095845 | up | 1.3075889 |
| chr11_22039 | 0.04856056 | up | 1.2086188 |
| chr11_22252 | 0.042011485 | up | 1.1441902 |
| chr1_2085 | 0.008808163 | up | 1.323964 |
| chr1_2177 | 0.009133823 | up | 1.1162066 |
| chr12_24683 | 0.034499902 | up | 1.2844226 |
| chr1_3145 | 0.03571804 | up | 1.3120632 |
| chr17_30153 | 0.002610224 | up | 1.5514717 |
| chr17_30221 | 0.018739423 | up | 1.2307092 |
| chr20_33733 | 0.010249853 | up | 1.4047002 |
| chr2_4963 | 0.005583577 | up | 1.2988802 |
| chr2_5243 | 0.005610816 | up | 1.4358892 |
| chr3_8038 | 0.009239781 | up | 1.3763573 |
| chr5_12617 | 0.038660917 | up | 1.2945753 |
| chr6_13311 | 0.047498155 | up | 1.4294362 |
| chrX_35230 | 0.02677866 | up | 1.4877636 |
| chrX_35618 | 0.008659811 | up | 1.4124161 |
